# EvAn: Neuromorphic Event-Based Sparse Anomaly Detection

**DOI:** 10.3389/fnins.2021.699003

**Published:** 2021-07-29

**Authors:** Lakshmi Annamalai, Anirban Chakraborty, Chetan Singh Thakur

**Affiliations:** ^1^Defence Research and Development Organisation (DRDO), Bangalore, India; ^2^Department of Electronic Systems Engineering, Indian Institute of Science (IISc), Bangalore, India; ^3^Department of Computational and Data Sciences, Indian Institute of Science (IISc), Bangalore, India

**Keywords:** neuromorphic camera, anomaly detection, event data, silicon retina, sparse

## Abstract

*Event-based* cameras are bio-inspired novel sensors that asynchronously record changes in illumination in the form of events. This principle results in significant advantages over conventional cameras, such as low power utilization, high dynamic range, and no motion blur. Moreover, by design, such cameras encode only the relative motion between the scene and the sensor and not the static background to yield a very sparse data structure. In this paper, we leverage these advantages of an event camera toward a critical vision application—video anomaly detection. We propose an anomaly detection solution in the event domain with a conditional Generative Adversarial Network (cGAN) made up of sparse submanifold convolution layers. Video analytics tasks such as anomaly detection depend on the motion history at each pixel. To enable this, we also put forward a generic unsupervised deep learning solution to learn a novel memory surface known as Deep Learning (DL) memory surface. DL memory surface encodes the temporal information readily available from these sensors while retaining the sparsity of event data. Since there is no existing dataset for anomaly detection in the event domain, we also provide an anomaly detection event dataset with a set of anomalies. We empirically validate our anomaly detection architecture, composed of sparse convolutional layers, on this proposed and online dataset. Careful analysis of the anomaly detection network reveals that the presented method results in a massive reduction in computational complexity with good performance compared to previous state-of-the-art conventional frame-based anomaly detection networks.

## 1. Introduction

This paper focuses on anomaly detection using bio-inspired event-based cameras that register pixel-wise changes in brightness asynchronously in an efficient manner, which is radically different from how a conventional camera works. This results in a stream of events *e*_*k*_, where *e*_*k*_ = {*x*_*k*_, *y*_*k*_, *t*_*k*_, *p*_*k*_}, *x*_*k*_ and *y*_*k*_ being the *x* and *y* coordinates of the pixel where an intensity change of pre-defined threshold has occurred, and *t*_*k*_ and *p*_*k*_ are the time (accurate to microseconds) and polarity ∈ {+1, −1} of the change, respectively. The asynchronous principle of operation endows event cameras (Delbruck and Mead, 1989; Posch et al., 2008; Delbruck and Barranco, 2010; Serrano-Gotarredona and Linares-Barranco, 2013) to capture high-speed motions (with temporal resolution in the order of μ*s*), high dynamic range (120−140*db*), and sparse data. These low latency sensors have paved the way for developing agile robotic applications (Annamalai et al., 2019), which was not feasible with conventional cameras. Only limited achievements have been accomplished in designing robust and accurate visual analytics algorithms for the event data.

Video anomaly detection (Joo and Chellappa, 2006; Miguel and Martinez, 2008) is a pervasive application of computer vision with its widespread applications as diverse as surveillance, intrusion detection, etc. However, the definition of anomalous activities is too generic and varies from scenario to scenario. Generally, the activities that occur with low and high probability are considered anomalous and normal activities, respectively. However, the definition of normal and anomaly is highly subjective, dependent on the task at hand. This paper introduces the stationary background and pedestrian walking as normalcy and activities such as running, bending, falling, fighting, and vehicles on the road as anomalies.

Anomaly detection in vision context requires fast response, which depends on the sensor's sensing capability and the computational complexity of vision algorithms. This paper proposes an anomaly detection solution using the event camera to address low latency in sensing modality. We start by putting forth the advantage of event camera sensing modality in anomaly detection application: (i) event camera comes with the ability to encode motion information at the sensor level and provides automatic background subtraction for stationary camera surveillance and (ii) yet another promising feature of event camera for anomaly detection is data sparsity, which is more pronounced especially when the camera is stationary. Given the sensing modality difference between event camera and conventional camera, we propose a solution that takes advantage of the data sparsity and the motion information encoded in the data, making our approach appropriate to sensor choice, suitably addressing the reduced computational complexity.

The rarity of anomalous activities makes it infeasible to construct anomaly detection as a two-class classification problem. Hence, our anomaly detection approach is posed as a generative deep learning (DL) problem. We formulate the task at hand as follows: (i) Given a set of normal activities, the network learns to fit a density model to predict the future activity, and (ii) when an anomaly occurs, the network will fail to predict future, which could be taken as an indication of the anomaly. One of the most exciting capabilities of the event camera is its ability to produce sparse data. The main benefit of this is that it allows us to build computationally less complex algorithms. To address this, we propose a sparse convolutional cGAN. In event domain, cGAN and GAN were proposed by Wang et al. (2019) and Robey et al. (2021) for the conversion of events to a high-quality image and to construct a neuromorphic version of CIFAR-10, respectively. However, the potential of cGAN built with sparse convolution layers has not been explored in this domain. Sparse convolutional cGAN allows detecting anomalies with much less computational complexity, which would not have been feasible otherwise.

Video analytics tasks such as anomaly detection involve an understanding of temporal information and spatial information to predict future activities. The temporal feature learning is usually guaranteed by adding an optical flow constraint or 3*D* convolution networks or temporal modeling networks such as long short term memory (LSTM). However, with the event camera, each pixel's motion history could be extracted into Time Surfaces (TS) (Gallego et al., 2019) or Memory Surface (MS) from the time information encoded for each event. TS and MS will enable us to circumvent computationally complex optical flow estimation or 3*D* convolution used in most state-of-the-art frame-based anomaly detection networks to capture motion features explicitly. State-of-the-art MS or TS, popularly known as motion history images, belongs to feature engineering, which mandates domain expertise and parameter tuning. This paper introduces an unsupervised shallow encoder–decoder architecture to learn a better sparse event MS. Proposed MS referred to here as DL memory surface embeds the rich motion history at each pixel individually. Although recently end-to-end learning of creating grid event representation of event data has been proposed in Gehrig et al. (2019) and Ciccone et al. (2020), it is tied to the task loss at hand. However, in memory surface generation, the advantages of unsupervised DL to learn nested concepts from data have not been explored. Unsupervised learning decouples the learning of memory surfaces and the higher level vision task, which is essential in scenarios where the labeled event data are scarce. This decoupling enables the proposed sparse DL memory surface to be applied to any video analytics task.

There has not been any attempt to create an event-based anomaly detection dataset. To bridge this gap and validate our algorithm's efficacy, we introduce a novel anomaly detection event dataset. The dataset was recorded from a type of event camera known as Dynamic Active Pixel Vision Sensor (DAVIS) (Lichtsteiner et al., 2008; Moeys et al., 2017) and also assimilated from publicly available pedestrian detection and action recognition event datasets. The dataset used for anomaly detection is highly challenging as there is lot of overlap in the statistics of our normal and anomalous activities. This overlap has been substantiated with statistical analysis performed on the dataset. We believe that the dataset and the proposed algorithm will have a new future in the event domain.

**Contributions:** In the context of the previous discussion, our contributions in this paper can be summarized as follows:

1. Memory surface generation: Generic DL-based data dependant unsupervised sparse memory surface generation.
2. Anomaly detection network: cGAN built with sparse submanifold convolution layers (low computational complexity).
3. Anomaly detection dataset.

## 2. Related Work

In this section, we review previous work in the areas of event memory surface generation and conventional frame-based anomaly detection.

### 2.1. Event Memory Surface Generation

Neuromorphic system processing should be essentially different from the conventional processing systems (Thakur et al., 2018). A popular methodology followed in literature is an adaptation of event data to make them compatible with conventional networks. A survey of event representations has been provided in Gallego et al. (2019). Recently, Sironi et al. (2018) proposed an exciting approach to encode time information by introducing a representation (highly resistant to noise) known as MS that exponentially weighs the information carried by past events. Following this, Zhu et al. (2019) has proposed an event representation by discretizing the time domain. However, this representation might result in higher computational costs when applied to a deep network. Calabrese and Easthope (2019) have generated frames by accumulating a constant number of events, thus claiming to have an adaptive frame rate.

In Park and Cho (2016), time-stamp maps are created using three distinctive techniques, pixel replication, temporal interpolation, and spatiotemporal correlation. These time-stamp maps are merged temporally for further processing, hence tending to lose the event camera's time information.

In Lagorce et al. (2017), the intensity image has been coded with the time stamp of each pixel (*x, y*) of recent positive and negative events in the given integration time *T* and around a spatial location of *R*×*R*. This image was further used to construct features recognized as time surfaces. Following this, Zhu et al. (2018) encode the first two channels as the number of positive and negative events that have occurred at each pixel and the last two channels as the time stamp of the most recent positive and negative events. This representation discards all the other time information except that of the recent event. Moreover, this kind of encoding is susceptible to noise.

Mitrokhin et al. (2019) and Alonso and Murillo (2019) have attempted to improve the time channel information by expertly combining the time information. In Mitrokhin et al. (2019), third channel stores average of the time stamp of the events that occurred at pixel (*x, y*) in a given temporal window of size δ*t*. Alonso and Murillo (2019) improved it by allocating four channels that encode the standard deviation of the time stamp of positive and negative events (separately) that happened at that specific pixel in the given time interval δ*t* in addition to their average value.

Earlier works restrained themselves from encoding basic information such as polarity. In Nguyen et al. (2017), a list of events is converted into images by recording the occurrence of the recent event in the given time. The drawback of this representation is that it encodes the latest event information solely at each pixel value. In Maqueda et al. (2018), a two-channel event image is created with histogram *h*^+^ and *h*^−^ of positive and negative events, respectively. Storage of different polarity events in different channels avoids the cancellation of events of opposite polarity at a given location. This choice proves to be better than that of Nguyen et al. (2017). The predominant setback of the above basic strategies is that they discard the treasured time information obtained from event cameras.

The alternative models that can cope with event data are biologically inspired neural networks acknowledged as spiking neural networks (SNN) (Russell et al., 2010). However, SNN has not become increasingly popular due to the lack of scalable training procedures. Although native training on an SNN is very challenging to scale, the shadow training methods proposed by Rueckauer et al. (2017) in the SNN toolbox have shown to scale to quite deep networks. For example, a 19-layer autoencoder was trained using the SNN toolbox in reducing latency in a converted spiking video segmentation network presented in Chen et al. (2021).

Memory surface representations mentioned earlier commonly use handcrafted kernels such as the alpha kernel or exponential kernel. In Gehrig et al. (2019), authors have proposed Multi-Layer Perceptron (MLP) with two hidden layers with 30 nodes each to implement a trilinear filter that produces a voxel grid of features. The events are considered as individual elements, thus discarding the temporal conditional dependence of the event sequence. In Ciccone et al. (2020), authors have proposed a matrix of LSTM cells to learn the mapping from events to 2*D* grid representation. However, the formulations in Gehrig et al. (2019) and Ciccone et al. (2020) are supervised end-to-end learning, which is task dependant and tuned to the particular task for which it was trained.

This paper proposes an unsupervised domain motivated memory surface learning that is data dependant but not task dependant. Note that, unlike (Gehrig et al., 2019; Ciccone et al., 2020), ours is entirely unsupervised and can be used as a general memory surface generation network for any motion analytic related vision task. Unlike (Gehrig et al., 2019; Ciccone et al., 2020), our DL memory surface generation can be utilized in scenarios where labeled data is critically low, which is not uncommon in event domain.

### 2.2. Anomaly Detection on Conventional Camera

As there is no prior work on event data anomaly detection, we briefly describe the frame-based DL algorithms for anomaly detection (Kiran et al., 2018). Researchers build a statistical model (reconstruction modeling and predictive modeling) to characterize the normal samples, and the actions that deviate from the estimated model are identified as anomalies.

Reconstruction modeling (Ng, 2011; Hasan et al., 2016; Sabokrou et al., 2016; Chalapathy et al., 2017; Chong and Tay, 2017) usually trains a deep auto-encoder type neural network to memorize the training videos so that they reconstruct normal events with lesser reconstruction error. Deep network's learning capability and generalization are too high that they do not conform to higher reconstruction error expectations for abnormal events. This led to the new attractive phase of predictive models, which is trained to predict the current frame based on past event's history. The frames which do not agree with the prediction are declared as anomalies. Researchers have contributed a lot toward predictive modeling with convolutional LSTM (Medel, 2016; Medel and Savakis, 2016; Luo et al., 2017) and generative architectures. Convolutional LSTM learns the transformation required to predict the frames. Generative models such as variational auto-encoder (VAE) (Diederik and Kingma, 2014) and generative adversarial network (GAN) (Goodfellow and Pouget-Abadie, 2014) learn the probability distribution to generate the future from the history, which makes them ideal candidates for anomaly detection.

Schlegl et al. (2017) proposed AnoGAN toward anomaly detection, which is trained with a weighted sum of residual loss and discriminator loss. Residual loss, |*x* − *G*(*z*)|, is defined as dissimilarity between original image *x* and the image *G*(*z*) generated from random noise *z*. Discriminator loss is the dissimilarity between the intermediate features representation of the original image and the reconstructed image. Hence, the discriminator acts as a feature extractor, not a hard classifier. However, temporal information has been discarded in modeling anomalies.

Ravanbakhsh et al. (2017b) proposed an anomaly detection framework that tries to model the anomalies based on motion inconsistency as well. The framework consists of two cGAN networks, trained on cross channel tasks of generating future frame from the optical flow (Brox et al., 2004) and vice versa, respectively. During test time, the two discriminators identify abnormal areas that correspond to outliers based on the distribution learned by the discriminators during the training phase.

Similar architecture has been followed in Ravanbakhsh et al. (2017a). However, the significant difference lies in the methodology used to detect possible anomalies. This work utilizes generators to reconstruct optical images and frames at test time, which results in unstructured blobs for anomalous events due to the network's inability to reconstruct unseen abnormal events.

Liu et al. (2018) proposed a GAN solution to capture the appearance and motion-based anomalies by leveraging reconstruction loss, gradient loss, and optical flow loss in addition to the adversarial loss. The motion constraint is modeled as the difference between the optical flow of predicted frames and the original frame. Recently, Yan et al. (2018) has proposed a 3*D* convolutional GAN to capture temporal information for anomaly detection. However, 3*D* convolution increases the computational complexity of the network.

We argue that frame-based anomaly networks suffer from the weakness of explicitly modeling normal activity with deep and dense convolutional architectures. The use of dense architectures renders their direct application on event data debatable, as the latter exhibits high data sparsity. In this work, we have proposed cGAN made up of sparse submanifold convolutional layers. The proposed network can realize the accuracy of frame-based DL networks while leveraging the event camera's sparse data structure, thus resulting in very low computational complexity.

## 3. Proposed Anomaly Detection Method

**Figure 1:**
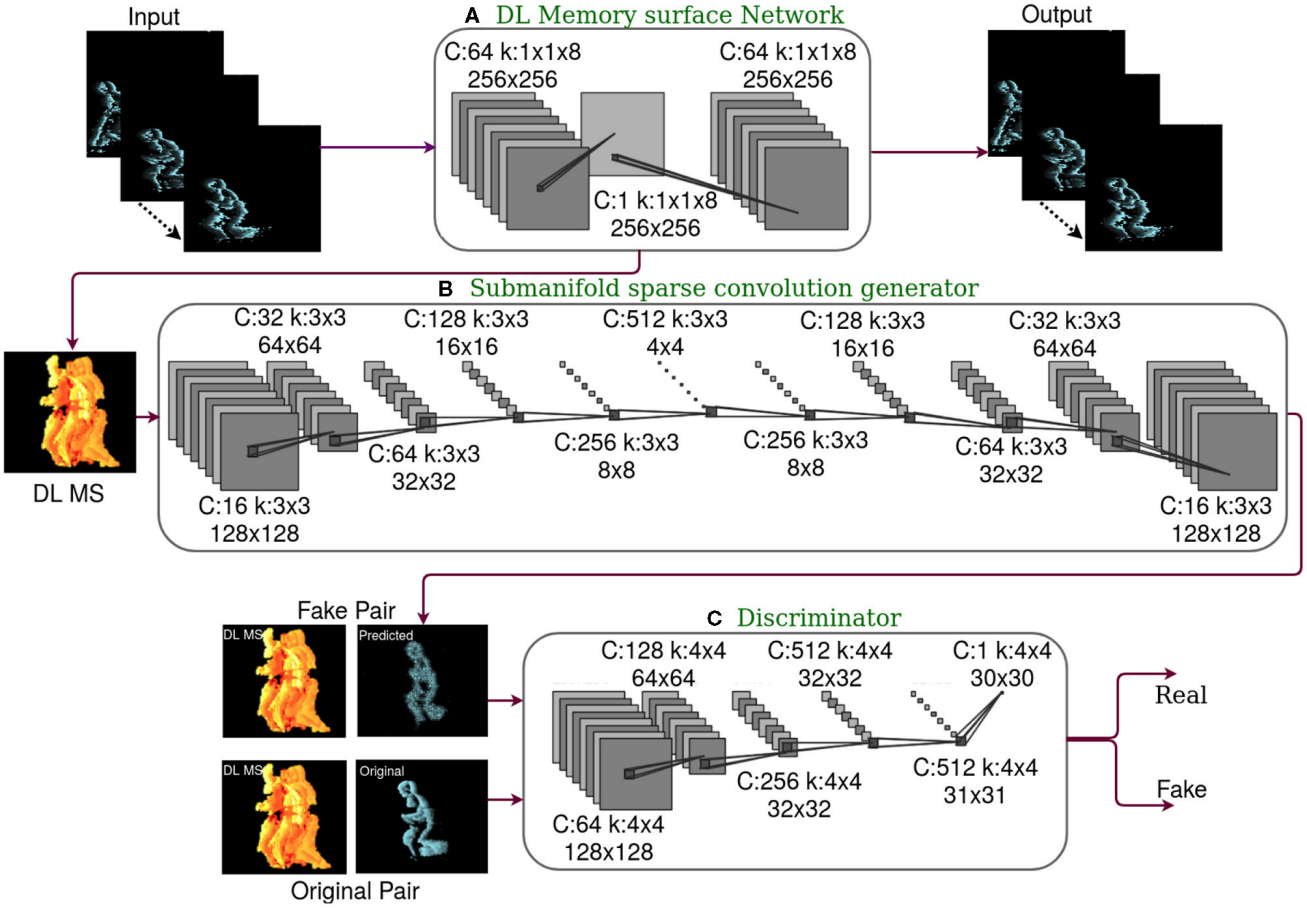
The framework of the proposed deep learning (DL) memory surface network and sparse convolutional conditional Generative Adversarial Network (cGAN), where C is the number of channels and k is the kernel. **(A)** DL memory surface network with event volumes as input and output. Trained as stand-alone network in an unsupervised setting with activation regularization. Sparse DL memory surfaces are generated at the bottleneck layer. **(B)** The generator of cGAN is built with Submanifold Sparse Convolution (SSC) layers, which predict future Time Surfaces (TS) conditioned on DL memory surface. Only generator is used during inference. Hence building a generator with SSC layers aids in the reduction of computational complexity. **(C)** Discriminator of cGAN, which is exposed to real pair of images (DL memory surface and original future TS) and fake pair of images (DL memory surface and predicted future TS). This contributes to the adversarial loss of cGAN during the training stage.

The pipeline of our event data prediction framework for anomaly detect ion is shown in Figure 1. Our methodology presents anomaly detection as a conditional generative problem that predicts future activity conditioned on past activities. The proposed cGAN network is constructed from sparse submanifold convolution layers to leverage the sparse nature of event data, thus reducing computation latency. We train a sparse convolutional cGAN to predict future TS Zhu et al. (2019) conditioned on the current DL memory surface. TS is formed by accumulating time stamp of events for a duration of 50*ms*. As the event camera data have noise effects, we have done preprocessing to remove the same. No information was retained after noise removal when Δ*T* was small. Hence, we have chosen Δ*T* = 50*ms* to have an optimum trade-off between temporal latency and information content. DL memory surfaces are generated by a DL memory surface generation network (details of which are furnished in the forthcoming sections). DL memory surface capture the time information encoded in the given set of events {*x*_*i*_, *y*_*i*_, *p*_*i*_, *t*_*i*_}_*t*_*i*_∈*T*_ into a single 2*D* structure known as DL memory surface.

We have also provided a theoretical explanation of the working of DL memory surface, cGAN, and the difference between general and sparse convolution in the Supplementary Material.

### 3.1. Architecture of Sparse Convolutional cGAN Network

The framework adopted here is a sparse convolutional cGAN architecture with a sparse convolutional generator, and conditional discriminator made up of convolution-BatchNorm-ReLU. The input and output differ in time instants, though they belong to the same underlying structure. Generator and discriminator architectures are designed around this. We start this section with a brief review of cGAN architecture. cGAN is a two-player game wherein a discriminator takes two points *x* and *y* in data space and learns to emit high probability when *x* and *y* are samples from the data distribution. While a generator learns to map a noise vector *z* drawn from ℙ(*z*) and input sample *y* drawn from ℙ_*d*_(*y*) to a sample $\hat{x}=G\left(z,y\right)$ that closely resembles the data *x*. The learning happens by solving the following minimax optimization:

(1) $$\begin{array}{l} \underset{G}{\min}\;\underset{D}{\max}\;{𝔼}_{y\sim {\mathbb{P}}_{d}(y)}{𝔼}_{x\sim {\mathbb{P}}_{d}(x|y)}\log\left[D\left(x,y\right)\right]\\ \;+{𝔼}_{y\sim {\mathbb{P}}_{d}(y)}{𝔼}_{\hat{x}\sim {\mathbb{P}}_{g}(x|y)}\log\left(1-D\left(\hat{x},y\right)\right] \end{array}$$

#### 3.1.1. Submanifold Sparse Convolution Generator

The generator comprises a sparse convolutional encoder-decoder architecture (middle row in Figure 1). The generator tries to predict future TS conditioned on the DL memory surface. GAN is generally a deep and computationally expensive architecture. In order to speed up inference, pooling layers have been added to CNN architectures with a decrease in spatial dimension and an increase in the number of channels as we go deep down the network. However, slow pooling is essential to retain spatial structures, particularly in sparse data. In order to offset this trade-off, the input data's sparsity should be utilized to reduce the computational complexity. Event cameras primarily respond to edges, thus resulting in spatial sparsity. In this paper, Submanifold Sparse Convolution (SSC) mentioned in Graham and van der Maaten (2017) is tuned to utilize the spatial sparsity encountered in the event data. Sparse layers result in reduced computation complexity. SSC convolutions compute convolution only on pixels termed as active sites. A pixel falls under the active site if the corresponding central site in the receptive kernel field is non-zero. On active sites, convolutions are carried out as Sparse Convolution (SC) Graham (2014).

We have used SSC to build our generator, whose layers are mainly responsible for the inference computation complexity. SSC is optimized to process sparse event data that lives in high-dimensional space. The blocks we have used in our encoder-decoder architecture of the generator are Minkowski layers (Choy et al., 2019) made up of Convolution/Deconvolution-Pooling-Activation. Activation functions are defined only on active sites. Deconvolution operations are defined as the inverse of the submanifold sparse convolution operation. The set of active sites is the same as that of the input to the corresponding SSC convolution layer.

We have used 6 convolution layers and 5 pooling layers in the encoder side with 6 deconvolution and 5 unpooling layers on the decoder side. The number of channels of encoder are 16, 32, 64, 128, 256, 512. Hence, the encoder produces 512 feature maps fed to the decoder to reconstruct the input by deconvolving and unpooling in reverse order of size and channels. A defining feature of the proposed architecture is SSC in the generator. Exhaustive implementation details of SSC can be found in Choy et al. (2019).

#### 3.1.2. Discriminator

Being motivated by the unique design of the discriminator proposed in Isola et al. (2017), we have used the PatchGAN classifier as a discriminator. Conventional discriminator classifies the entire time surface as real or fake. However, patchGAN discriminator is a convolutional network that can classify each tile of predicted and original future time surfaces as real or fake, respectively, conditioned on the DL memory surface. PatchGAN generates a *N*×*N* output array where *N* is the number of tiles into which the time surface is divided along rows and columns. The average of classification of tiles is considered to declare the entire time surface as real or fake. Working on individual tiles rather than on the entire time surface capture the details in sharp, high-frequency edges. PatchGAN discriminator works on any image size with the assumption that pixels separated by patch radius are independent.

### 3.2. DL Memory Surface Generation Network

As it has been mentioned previously, we have proposed an unsupervised learning based event data memory surface known as DL memory surface. DL memory surface is generated by a shallow, computationally inexpensive encoder-decoder network architecture (top row in Figure 1) with a loss function that includes a data term and a sparsity term. We start this section with an introduction to the architecture of the encoder-decoder network, followed by details on the loss function.

#### 3.2.1. Network Architecture

We adapt fully convolutional encoder-decoder architecture (Hinton and Salakhutdinov, 2006) that maps a discretized volume of event data to a single image known as DL memory surface *Ms* at the bottleneck layer. Discretized volume of event data (*Ev* = [*ev*_0_, *ev*_1_…*ev*_*B*_]) is produced by stacking events into TS (*ev*_*i*_) (Zhu et al., 2019), given a time duration *T* and a set of *B* discrete time bins [*b*_0_, *b*_1_…*b*_*B*_] each with Δ*T* duration.

The input and output are renderings of the discretized volume data *Ev*. Input event volume passes through the encoder convolution layer with 64 channels and 1 × 1 convolution, followed by bottleneck convolution (DL memory surface extraction layer) layer with a single channel. The non-linearity injected between the layers is ReLU, with activation regularization imposed on them. The convolution stride is 1 pixel in the spatial dimension. Spatial padding is fixed to retain the spatial dimension.

In order to model only the temporal history embedded in the data without upsetting the spatial distribution, it is adequate to restrict the convolution operation across the time dimension. Hence, we have designed the network with 1*D* convolution layers inspired by the paper of Lin et al. (2013), which was the first to implement 1 × 1 convolution. As we needed the network to learn temporal kernels that aids in accumulating event data over time, the defined 1*D* convolution is over the temporal dimension alone. As studies have shown, 1*D* CNN can learn complex tasks (Szegedy et al., 2015; Iandola et al., 2016) with shallow architecture, unlike its counterpart 2*D* CNN, thereby resulting in a “small” network.

#### 3.2.2. Loss Function for DL Memory Surface

The DL memory surface generation network attempts to learn a function *h*_θ_*MS*__(*Ev*) in such a way that the target values $\left[\hat{ev_{0}},\hat{ev_{1}}\ldots \hat{ev_{B}}\right]$ is similar to that of the input [*ev*_0_, *ev*_1_…*ev*_*B*_], while the bottleneck layer models the temporal information encoded by the event camera. The architecture consists of two parts, encoder and decoder defined by the transformation functions ϕ_θ_*E*__ : *Ev* → *Ms* and ψ_θ_*D*__:*Ms* → *Ev*, respectively, with θ_*D*_ and θ_*E*_ being the parameters of decoder and encoder, respectively. DL network discovered an interesting representation while preserving the input event data's sparse structure. The inclusion of 1*D* gives us the liberty to use shallow networks but still forces the network to learn more appropriate temporal information.

In order to maximize the usefulness of the latent variable encoding, we use a data term that tries to model the probability distribution {ℙ(*Ev*∣Ms*)} of getting the event discretized volume, *Ev*, given the ideal DL memory surface, Ms*, by maximizing the forward *KL* divergence between the ideal distribution ℙ(*Ev*∣Ms*) and our estimate $\mathbb{P}\left(Ev|\hat{Ms}\right)$ (Equation 2). Forward *KL* divergence will result in the best latent variable that covers all the models of probability distribution of normal videos.

(2) $$\begin{array}{l} KL={𝔼}_{Ev\sim \mathbb{P}\left(Ev|Ms^{*}\right)}\log\left[\mathbb{P}\left(Ev|Ms^{*}\right)\right]\\ \;-{𝔼}_{Ev\sim \mathbb{P}\left(Ev|Ms^{*}\right)}\log\left[\mathbb{P}\left(Ev|\hat{Ms}\right)\right] \end{array}$$

Since the first term does not depend on the estimated latent variable, it could be ignored. Hence, the second term of Equation (2) boils down to maximizing the log likelihood of $\mathbb{P}\left(Ev|\hat{Ms}\right)$ when the sample size tends to infinity. The output of the decoder can be modeled as a function of latent variable $\hat{Ms}$ and noise η ~ ℕ(0, 1) as $\psi_{\theta_{D}}\left(\hat{Ms}\right)+\eta$. This makes $\mathbb{P}\left(Ev|\hat{Ms}\right)$ a Gaussian distribution with mean $\psi_{\theta_{D}}\left(\hat{Ms}\right)$. Thus, maximizing the log likelihood turns out to minimizing $-∥Ev-\psi_{\theta_{D}}\left(\hat{Ms}\right){∥}^{2}$.

To preserve the sparsity in the event data at the bottleneck layer, we have introduced an activation regularization term in the loss function in addition to the data term. To utilize the advantages of *L*_1_ and *L*_2_ norms, the activation regularization term includes a combination of *L*_1_ and *L*_2_ regularization of activations in the bottleneck layer.

We have performed a thorough evaluation of our proposed DL memory surface generation network as a standalone architecture on event data and provided the same in Supplementary Material. The experiments include self-analysis and a thorough comparison with the existing state-of-the-art methods. Self-analysis includes the study on sparsity regularization parameters as well. Comparison comprises performance and computational complexity-related experiments.

## 4. Experimental Validation

This section evaluates different components of the proposed method on the dataset introduced in the forthcoming section. We have provided a complete analysis of the anomaly detection network made up of sparse sub-manifold convolutional layers. To emphasize the task-independent utility of our DL memory surface, we have also provided experiments to analyze the same on a different vision application in the Supplementary Material. We start this section with a description of the dataset.

### 4.1. Dataset

**Figure 2:**
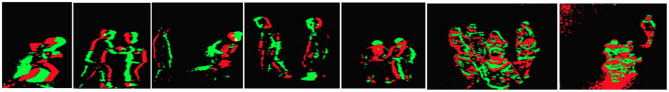
Display of few samples of event frames from the dataset. Event frames are formed with recent on and off events that occur in a specified time window. On and Off events are pseudo-colored as red and green, respectively.

We validated our algorithm on the proposed dataset assimilated from three different types of datasets, two datasets generated and introduced in Shu et al. (2019) targeted for pedestrian detection, and action recognition and a novel dataset recorded by DAVIS tailored for anomaly detection. Few samples from the datasets used are displayed in Figure 2. The complexity of the dataset considered is analyzed in terms of the event rate of normal vs. anomaly.

#### 4.1.1. Dataset Available Online

Previously, event-based datasets were made available for other vision-based tasks such as visual odometry (Mueggler et al., 2017) and object recognition (Li et al., 2017). Pioneering work from Shu et al. (2019) has introduced open-access datasets for motion-related tasks such as pedestrian detection and action recognition recorded with DAVIS sensor with a resolution of 346 × 260. Only events, not APS data, were recorded in order to save the storage space. As there is a significant change in viewpoint, it is conceivable to include data from the pedestrian detection dataset and the action recognition dataset. Furthermore, this will yield an opportunity to test the proposed algorithm under two different scenarios. Our formulation has carefully picked relevant samples from the action recognition (ActDataset) dataset and pedestrian detection dataset (PedDataset). The details of the activities considered as normal and anomalous are provided in Table 1.

**Table 1:** Statistics of AnoDataset, ActDataset (Shu et al., 2019), and PedDataset (Shu et al., 2019) used for evaluating the proposed algorithm.

**Actions**	**AnoDataset**	**ActDataset**	**PedDataset**	**Clips**
**Normal**				
Walking	✓	✓	✓	50
**Anomaly**				
Running	✓			10
Fighting	✓			4
Bending	✓	✓		43
vehicle			✓	2
Falling down		✓		30
Sitting	✓	✓		30
Arm cross		✓		30
Get Up/Sit		✓		30
Picking up		✓		30
Throwing		✓		30
Jumping		✓		30
Kicking		✓		30
Turning		✓		30
Tying		✓		30

#### 4.1.2. Dataset Recorded for Anomaly Detection

AnoDataset is our newly collected dataset as proposed in this paper, which was recorded for anomaly detection. This new anomaly dataset will benefit the neuromorphic community. We present two variations of the event dataset from two distinctive sets of environments, an indoor empty lab environment and an outdoor corridor environment, to set a realistic baseline for algorithm evaluation. This dataset consists of short event clips of pedestrian movements parallel to the camera plane, captured from a static DAVIS camera with a resolution of 346 × 260. Each recording lasts about 30 s on average. The normal and anomalous scenes are staged with typical training videos consisting of people walking. The testing videos consist of a variety of anomalous activities summarized in Table 1.

#### 4.1.3. Analysis of Dataset

**Figure 3:**
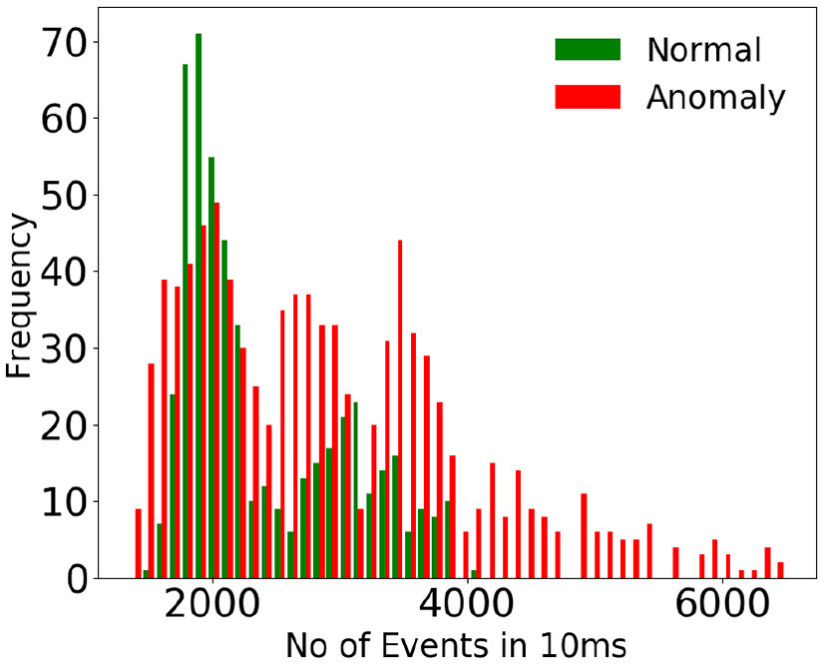
Display of histogram of event rate of normal vs. anomalous activity. The event rates are estimated for a standard time interval of 10 ms. Huge overlap in the histogram of event rate indicates that the anomalous activities considered do not differ from normal activity in terms of rate of motion.

**Figure 4:**
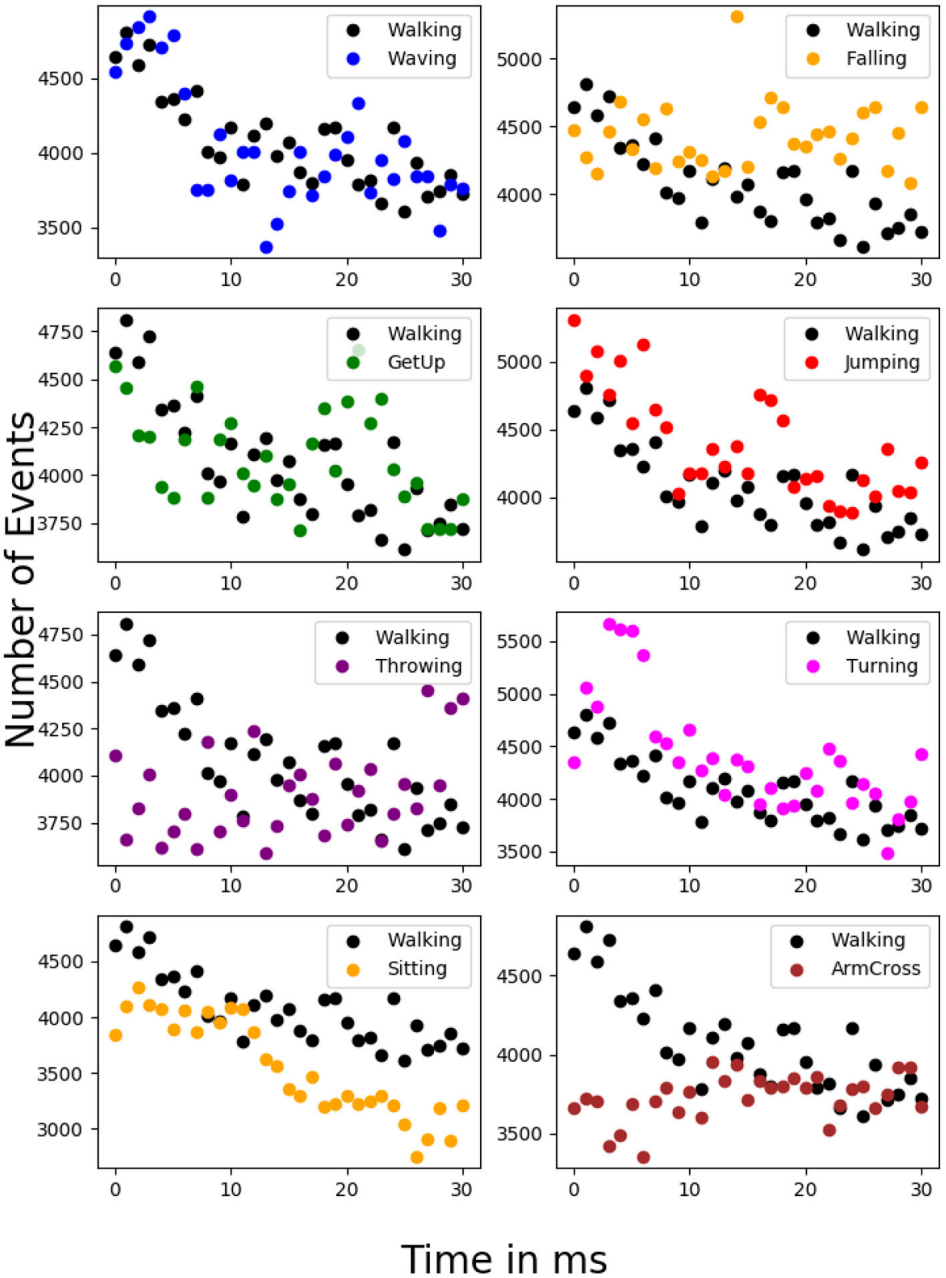
Display of event rate of normal vs. anomalous activity at different time instants for a standard time interval of 50 ms. Huge overlap in the event rate indicates that the anomalous activities considered do not differ from normal activity in terms of rate of motion.

To emphasize the complexity of the dataset considered, we have estimated the event rates of various anomalous activities vs. normal activity at various time instants for a uniform time interval. Figure 3 shows the histogram of event rates (for 10 ms) for normal and anomalous activity, and Figure 4 displays the number of events (for 50 ms) vs. various time instants for normal and anomalous activities. It could be seen that there is much overlap between normal and anomalous activities in terms of event rate, which indicates that the rate of motion of the normal and anomalous activities resemble each other, which justifies the use of a deep network to detect anomalies.

### 4.2. Implementation Details

The input discretized event volume, formed by accumulating events, is passed through a 3*D* convolution layer, which has a receptive field of 1 × 1 × *C*, where *C* is the number of channels. DL memory surface network, initialized with random weights sampled from a standard normal distribution, is trained with these input event volumes to reconstruct its inputs. The activation regularization of the DL memory surface network is fixed for anomaly detection network experiments. We extract the DL memory surface from the bottleneck layer of the DL memory surface network. We used a 2*e*^−4^ learning rate and 0.5 momentum.

A sparse convolutional cGAN anomaly detection network is trained with normal activities to predict future TS conditioned on DL memory surface. The generator's architecture is a sparse convolutional encoder-decoder, and that of the discriminator is a classifier architecture, the details of which are provided in previous sections. DL memory surface is fed to the generator network as a sparse tensor whose value is defined only at discrete locations specified by the indices matrix. Sparse tensor representation is crucial to saving memory space and maintaining sparsity in the generator network's computation. The optimization technique used is Stochastic Gradient Descent (SGD) with a learning rate of 0.1 and momentum of 0.9.

### 4.3. Evaluation Metrics

The various criteria used to evaluate the proposed anomaly detection network are recall, precision, F1 score, and accuracy as given below,

(3) $$\begin{array}{l} \;Recall=\frac{TPR}{TPR+FNR}\\ \;Precision=\frac{TPR}{TPR+FPR}\\ \;F1=\frac{2*precision*recall}{precision+recall}\\ Accuracy=\frac{TPR+TNR}{TPR+TNR+FPR+FNR} \end{array}$$

All the measures used are based on True-Positive Rates (TPR), False-Positive Rates (FPR), True-Negative Rates (TNR), and False-Negative Rates (FNR), where positive and negative denote the presence and absence of anomalous activities. The recall is the classifier's ability to recall positive classes. The ratio between the true-positive rate and the total number of retrieved images is referred to as precision. F1-measure indicates the balance between precision and recall. The fraction of accurate predictions of the model is termed accuracy. In addition to this, the Equal Error Rate (*EER*) is also used to summarize the anomaly detection network's performance. *EER* is the ratio of frames that are misclassified at *FPR* = 1 − *TPR*.

### 4.4. Experiments and Results on Self-Analysis

This section evaluates the underlying claim of the sparse convolutional anomaly network proposed. The claim is that given a sequence that contains normal and anomalous activities, the network automatically makes the anomalous activities stand out, distinguished from the normal scenarios. In order to achieve this, the network must have learned to predict normal activities but not anomalous activities. This section provides experimental results to substantiate this capability of the proposed anomaly network.

#### 4.4.1. Prediction Capability of Anomaly Network

**Figure 5:**
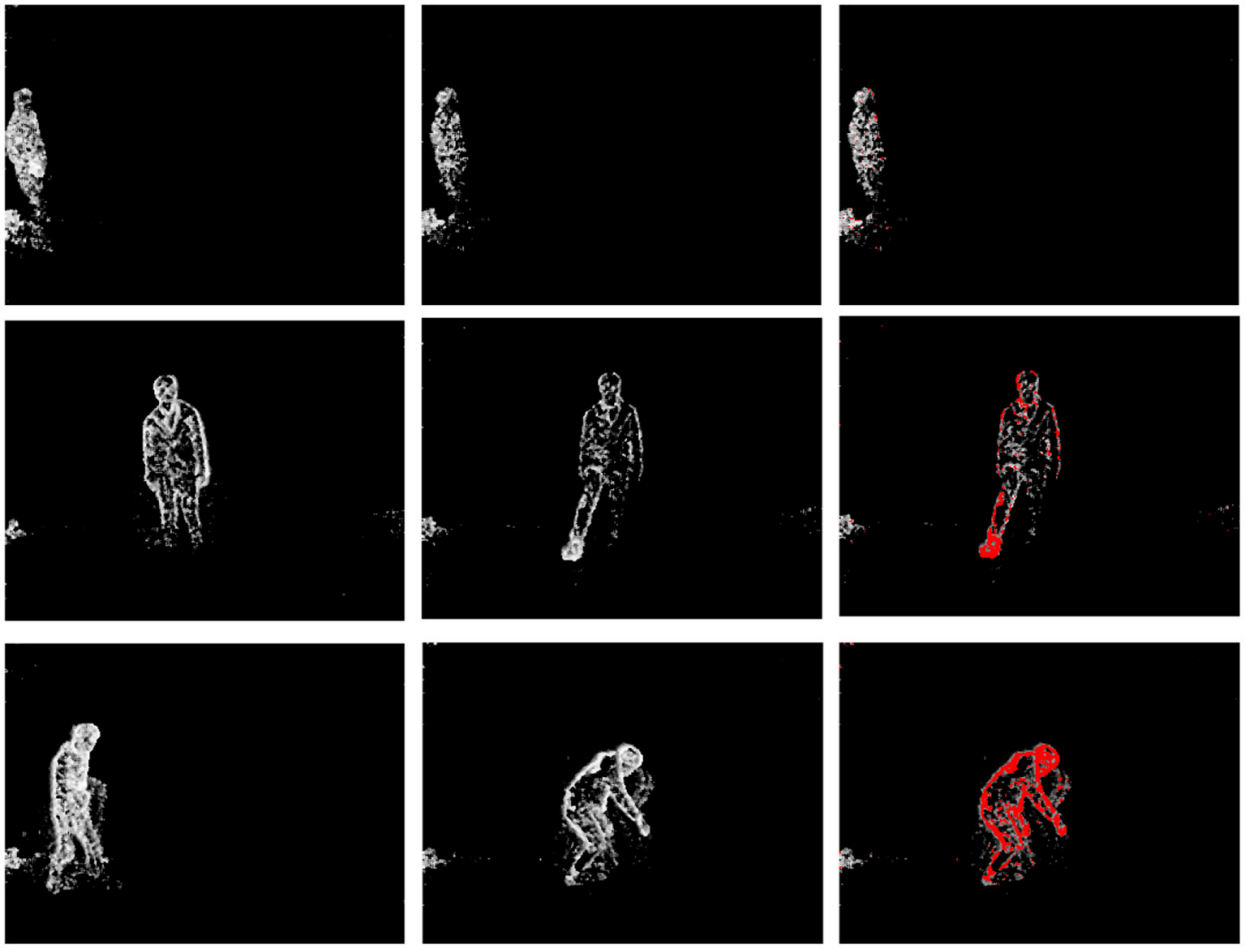
**Top**: Walking (normal) activity, **Middle**: Kicking (anomalous) activity, **Bottom**: Picking (anomalous) activity. Visualization of original events (left and middle) at times *t* − 1 and *t* and the difference between original (*en*^*t*^) and predicted events $\left({\hat{en}}^{t}\right)$ overlaid as red over the original events if it exceeds a pre-defined threshold (right) $\left(|{en}^{t}-\left({\hat{en}}^{t}\right)|\geq Threshold\to red\right)$. It could be seen that the difference between original and predicted events (red color) is prominent in the case of anomalies.

The proposed approach assumes that given that the network is trained with normal activities, it should have learned to predict the future TS in the case of normal activities and fail to do so in the case of anomalous occurrences. To validate that the goal mentioned above is achieved, we have subjected the network trained on normal activities to predicting future TS. Figure 5 provides visualization of original TS (left and middle column) at *t* − 1 and *t*, and color (red) coded thresholded difference (right column) between the original (*en*^*t*^) and predicted $\left({\hat{en}}^{t}\right)$ TS embedded in the original TS $\left(|{en}^{t}-\left({\hat{en}}^{t}\right)|\geq Threshold\to red\right)$. Top row in Figure 5 displays the experiment results of normal activity, whereas middle and bottom rows in Figure 5 display the results of anomalous activities. This emphasizes the fact that the network has learned to predict future TS of normal activities. In the case of anomalous activities, the experiments illustrate the good anomaly localization capability of the network.

#### 4.4.2. MSE Plots of Normal-Anomalous Sequences

**Figure 6:**
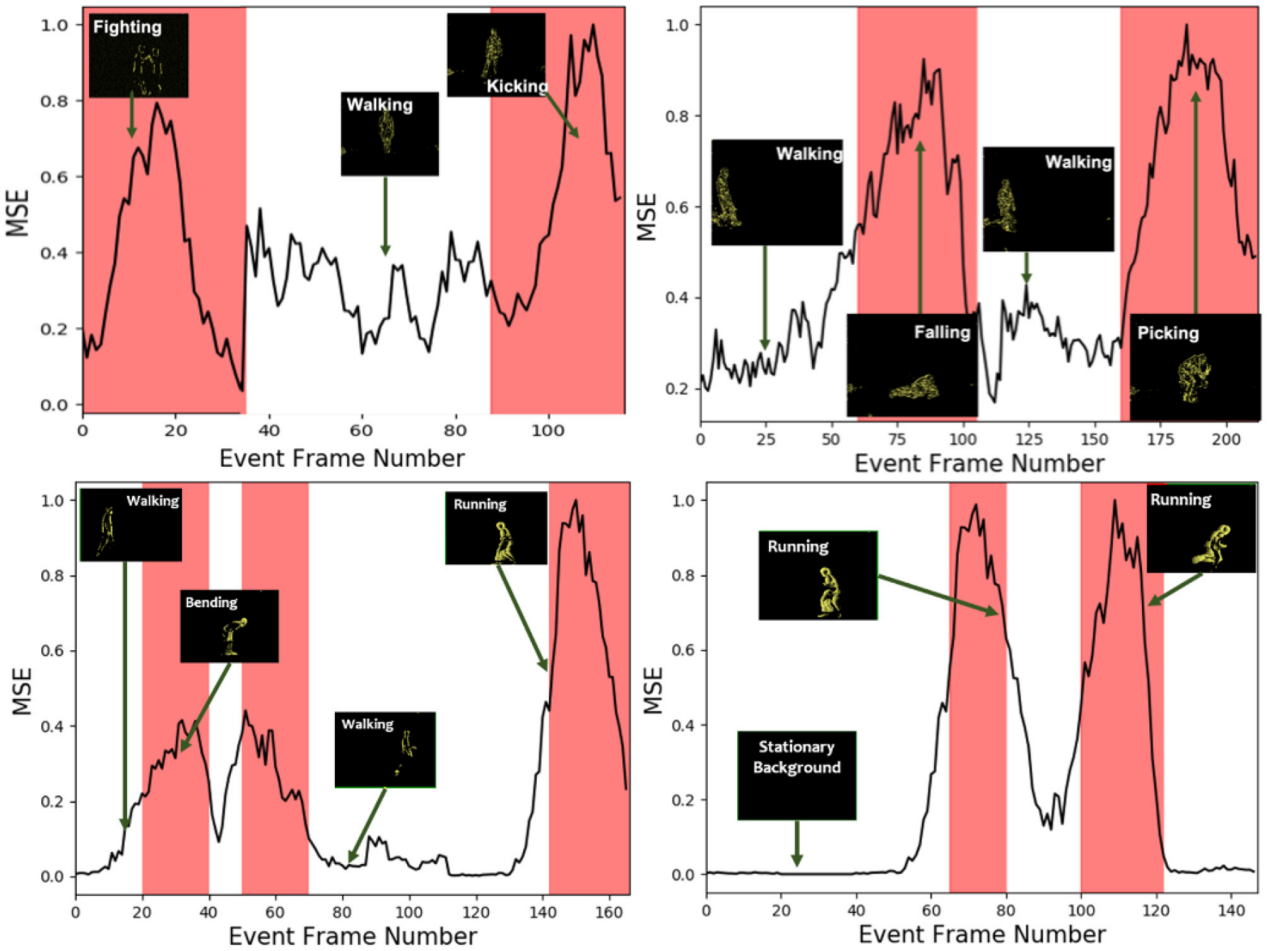
Visualization of mean square error (MSE) between original and predicted Time Surfaces (TS). White color corresponds to a time frame of normal activity, and red corresponds to abnormal activity. **Top left**: Walking is a normal activity with kicking and fighting as anomalies. **Top right**: Walking is a normal activity, with falling and picking as an anomaly. **Bottom left**: Walking as normal with bending and running as an anomaly. **Bottom right**: Stationary background as normal with running as an anomaly. MSE is high during anomalous activities as the anomaly network cannot predict the future TS in those cases as it has not seen them during training.

To quantitatively evaluate the anomaly detection network performance, testing cases with intermittent abnormal activities from the datasets mentioned above were presented to the network. In this experiment, the proposed algorithm detects anomalous activities at the event TS level by estimating prediction error. The prediction error is evaluated as normalized mean square error (MSE) between the TS predicted by the generator network of sparse Convolutional cGAN and the ground truth TS. MSE is estimated as given in Equation (4), where $\hat{en_{xy}}$ and *en*_*xy*_ are predicted, and original normalized time stamps (values between 0 and 1) at the location (*x, y*) in the TS and *N*_*x*_ and *N*_*y*_ are the number of rows and cols, respectively. It should be noted that the co-occurrence of the spatial location of anomaly is not considered for evaluation. Figure 6 shows the plot of the MSE vs. event frame number. It could be seen that event data with normal sequences have lower reconstruction error than that of events that belong to anomalous events as the network does not have enough knowledge to predict anomalous events.

(4) $$MSE=\frac{1}{N_{x}N_{y}}\sum_{x=1}^{N_{x}}\sum_{y=1}^{N_{y}}\hat{en_{xy}}-en_{xy}$$

#### 4.4.3. Quantitative Evaluation of Anomaly Network

In this section, we quantitatively assess the performance of the proposed sparse convolutional anomaly network. This section brings out the analysis of the network's capability to predict abnormal activities. Abnormality detection is evaluated over a range of thresholds. Table 2 displays the results of the FPR (False Positive Ratio), TPR (True Positive Ratio), Precision, and Recall averaged over anomalies.

**Table 2:** Quantitative analysis of anomaly network in terms of False-Positive Rates (FPR) vs. True-Positive Rates (TPR) (left) and Precision vs. Recall (right) averaged over various anomalous activities.

**FPR**	**TPR**	**Precision**	**Recall**
1.0	1.0	0.315	1.0
0.666	0.765	0.538	0.0765
0.633	0.765	0.545	0.0765
0.306	0.750	0.658	0.750
0.135	0.663	0.850	0.639
0.078	0.639	0.948	0.616
0.0	0.543	1.0	0.220

#### 4.4.4. Quantitative Evaluation on Anomalies Similar to Normal Activity

**Figure 7:**
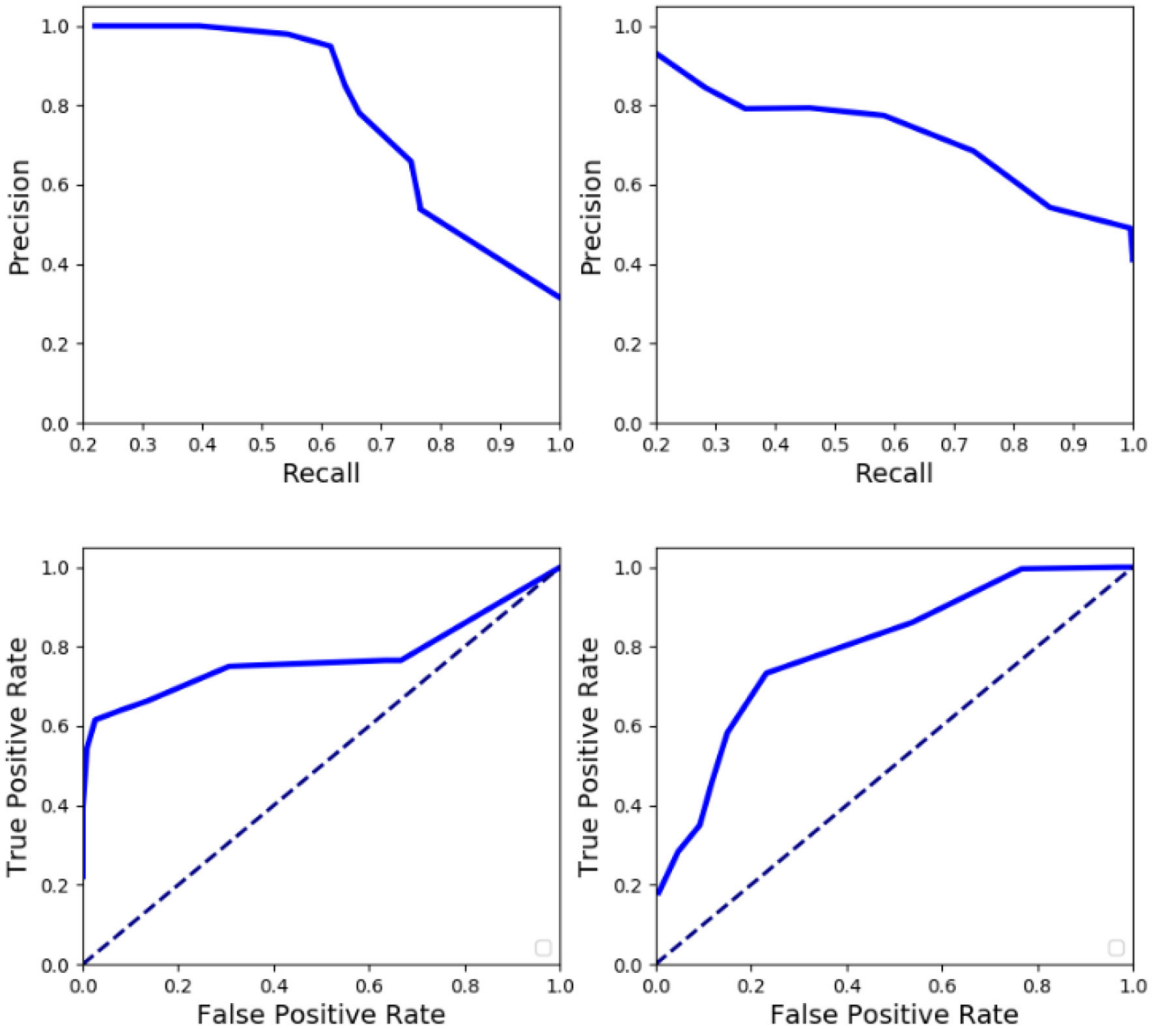
Quantitative analysis of anomaly network in terms of recall, precision, and ROC averaged over various anomalous activities (**Left**: Falling, GetUp, and Jumping anomalies; **Right**: Kicking, Picking, and Sitting anomalies). The difficult anomalies have been grouped together based on their level of overlap with normal activity.

We have also analyzed the sparse anomaly detection network on individual anomalies whose event rate overlaps with normal activities. Quantitative analysis in terms of AUC, EER, average precision, and F1 score is furnished in Table 3. Table 4 displays a detailed analysis of FPR and precision at different TPR on a sub-set of proposed anomalies, which are relatively challenging to detect. Anomaly detection was also evaluated over a range of thresholds to construct the ROC curve (FPR vs. TPR) and Precision-Recall (PR) curve on different anomalies. Figure 7 displays the results of the receiver operating characteristics (ROC) and PR (Precision-Recall) curve of the proposed method on various anomalies. It could be visualized that the proposed method performs well even for anomalies that do not differ from normal activities in terms of rate of motion.

**Table 3:** Quantitative analysis of proposed network in terms of Area Under Curve (AUC), Equal Error Rate (EER), average precision, and F1 score.

**Anomaly**	**AUC (%)**	**EER (%)**	**Avg precision (%)**	**F1 score (%)**
Falling	96	14	94	83
GetUp	93	18	88	75
Jumping	99	1	99	80
Kicking	83	24	82	56
Picking	82	23	78	72
Sit	82	25	74	87

**Table 4:** Quantitative analysis of proposed network in terms of True-Positive Rates (TPR), False-Positive Rates (FPR), and Pr (Precision).

**Falling**			**GetUp**			**Kicking**			**Picking**			**Sitting**		
**TPR**	**FPR**	**Pr**	**TPR**	**FPR**	**Pr**	**TPR**	**FPR**	**Pr**	**TPR**	**FPR**	**Pr**	**TPR**	**FPR**	**Pr**
1.0	1.0	0.33	1.0	1.0	0.28	1.0	1.0	0.29	1.0	1.0	0.47	1.0	1.0	0.46
0.92	0.29	0.60	0.95	0.4	0.48	0.8	0.56	0.37	0.98	0.79	0.52	0.98	0.9	0.49
0.89	0.18	0.70	0.77	0.10	0.73	0.76	0.16	0.65	0.93	0.31	0.72	0.8	0.4	0.68
0.86	0.07	0.84	0.72	0.05	0.84	0.64	0.02	0.94	0.28	0.05	0.8	0.50	0.26	0.73
0.78	0.02	0.93	0.68	0.0	1.0	0.48	0.0	1.0	0.2	0.0	1.0	0.14	0.02	0.85

#### 4.4.5. Computational Complexity

Table 5 shows the comparison of the computational complexity of dense and sparse layers in terms of FLOPs (Floating Point OPerations). FLOP is generally used as a metric to evaluate the computational complexity as it does not depend on the hardware and the implementation. *C*_*in*_ and *C*_*out*_ are the number of input and output channels for a particular layer, (*m, n*) is the size of the image, *k* is the size of the kernel, *N*_*a*_ is the number of active sites in the whole image, and $N_{k}^{2}$ is the number of non-zero pixels in the receptive field of the kernel. A particular pixel is active if it falls in the center of the kernel's receptive field. The number of multiplications and additions required for standard convolution for a particular pixel are $C_{out}\left(k^{2}C_{in}\right)$ and $C_{out}\left(k^{2}C_{in}-1\right)$, respectively.

**Table 5:** Comparison of the computational complexity of dense and sparse layers in terms of Floating Point OPerations (FLOPs).

**Layer**	**Dense**	**Sparse**
Convolution	$mnC_{out}\left(2k^{2}C_{in}-1\right)$	$N_{a}C_{out}\left(2N_{k}^{2}C_{in}-1\right)$
Max pooling	$mcC_{out}k^{2}$	*N*_*a*_ *C*_*out*_ *N*_*k*_
ReLU	*mnC*_*in*_	*N*_*a*_ *C*_*in*_

**Figure 8:**
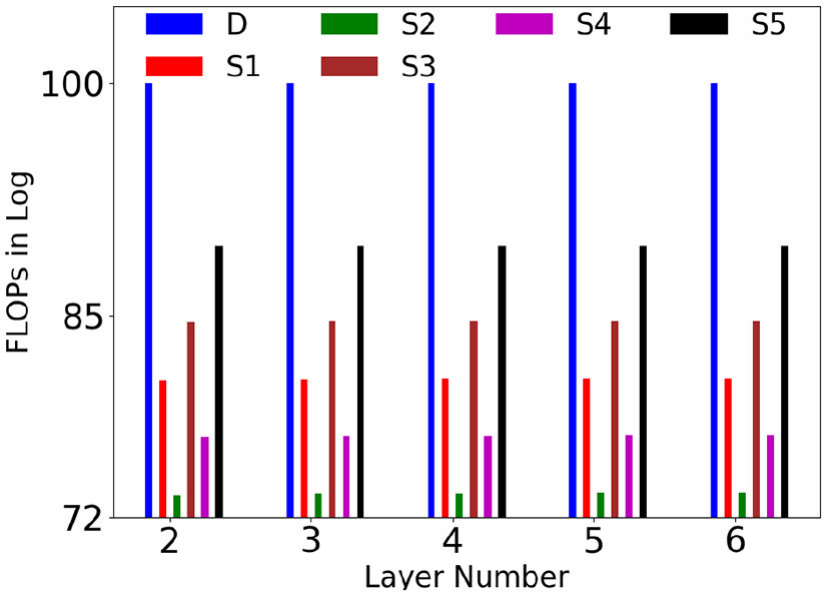
Comparison of the computational complexity of dense (D) and SSC layers (y axis is in log scale), S1 $\left(\frac{N_{a}}{mn}=0.25,N_{k}^{2}=1\right)$, S2 $\left(\frac{N_{a}}{mn}=0.0625,N_{k}^{2}=1\right)$, S3 $\left(\frac{N_{a}}{mn}=0.25,N_{k}^{2}=2\right)$, S4 $\left(\frac{N_{a}}{mn}=0.0625,N_{k}^{2}=2\right)$, S5 $\left(\frac{N_{a}}{mn}=0.25,N_{k}^{2}=5\right)$ at different levels in terms of FLOPs. For better visualization, FLOPs are converted into log and estimated as percentage of dense layer FLOP $\left(\frac{\log S_{i}}{\log D}*100\right)$.

We have given plot of comparison of computational complexity of dense and sparse layers at different levels at $N_{k}^{2}=1,2,5$ in Figure 8 ($N_{k}^{2}$ is taken as constant across all the layers. On an average, $N_{k}^{2}$ was varying from 1 to 5). For better visualization, FLOPs are converted into log domain and estimated as percentage of dense layer FLOP $\left(\frac{\log S_{i}}{\log D}*100\right)$, where *D* and *S*_*i*_ are dense and SSC layers. It could be noted that SSC layers requires far lesser computation than dense layers, especially when $\left(\frac{N_{a}}{mn}=0.0625,N_{k}^{2}=1\right)$.

### 4.5. Experiments and Results on Comparison With State of the Art

This section compares the proposed method with conventional frame-based approaches in terms of accuracy and computational complexity. As there is no existing anomaly detection network for event cameras, we compare our method with state-of-the-art frame-based anomaly detection approaches.

This section provides comparison of the proposed anomaly detection method with conventional frame-based algorithms (Hasan et al., 2016; Chong and Tay, 2017; Liu et al., 2018), Ftre (Schlegl et al., 2017), Gen (Schlegl et al., 2017), and (William et al., 2016). The details of the implementations used are summarized below.

AnoDet B (Chong and Tay, 2017) is a spatiotemporal auto-encoder architecture, which is made up of spatial and temporal autoencoders to learn spatial features and temporal patterns, respectively. The discretized volume of events mentioned in the earlier section of the paper is fed as input to the architecture. The event TS is classified as normal and anomalous based on the reconstruction error. We have adapted the code implementation provided at https://github.com/harshtikuu/Abnormal_Event_Detection.

AnoDet C (Hasan et al., 2016) uses a fully convolutional autoencoder in addition to conventional motion feature descriptors to learn low- and high-level features. The input is constructed as a discretized event volume of TS stacked together. The parameters are fixed as the implementation provided at https://github.com/NRauschmayr/Anomaly_Detection. The reconstruction error is estimated as the sum of per pixel error based on which an event TS is classified as normal or anomaly.

AnoDet F (William et al., 2016) is a deep recurrent convolutional neural network for future frame prediction. The implementation we have used is the architecture implemented as a custom layer in Keras https://github.com/coxlab/prednet. To predict future TS accurately, it needs a sequence of event TS as input to learn the objects' motion dynamics. An event TS is classified as normal or anomaly based on the prediction error.

AnoDet E (Schlegl et al., 2017) and AnoDet A (Schlegl et al., 2017) are the two versions of an anomaly detection network, which utilizes predicted feature and image, respectively, for anomaly detection. The architecture is made up of deep convolutional GAN, and it works on static images. The input is individual event TS formed by discretizing the events. It provides an anomaly score as a measure of fit of the predicted event TS to the original TS. The TensorFlow implementation is available at https://github.com/tSchlegl/f-AnoGAN.

AnoDet D (Liu et al., 2018) depends on the optical flow network to extract motion information for anomaly detection. Hence, it has been tested without training, which leads to deficient performance. Implementation is available at https://github.com/StevenLiuWen/ano_pred_cvpr2018.

#### 4.5.1. Classification Performance Analysis

**Figure 9:**
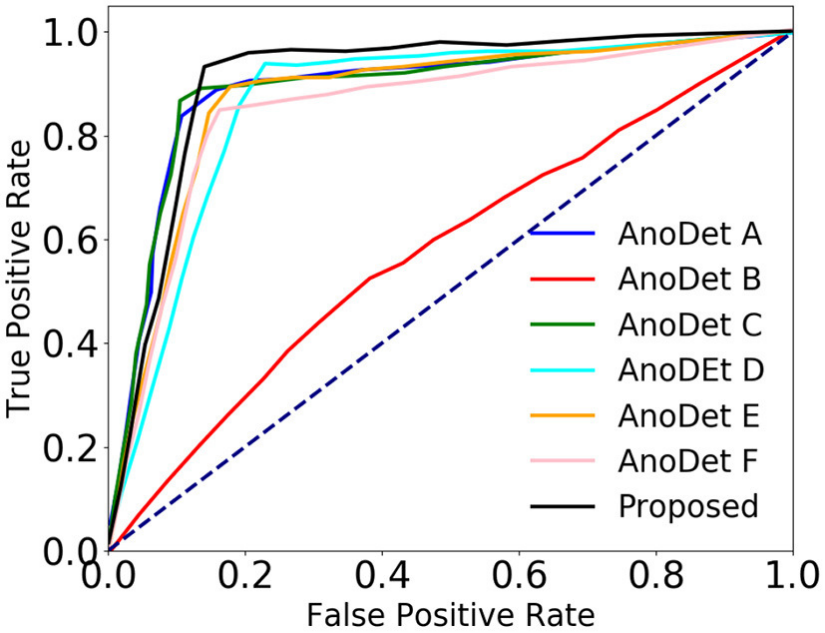
Comparison of the proposed anomaly network with state-of-the-art frame-based anomaly networks in terms of ROC.

As we compare methods based on AUC, EER, and ROC curves, the experiment is not sensitive to the threshold setting used to classify a particular event frame as normal or anomaly. It could be inferred from Table 6 that the proposed network performs better than the state-of-the-art frame-based anomaly detection networks. We have also provided the comparison of the proposed method with state-of-the-art methods in terms of ROC (Figure 9) generated from AUC and EER.

**Table 6:** Comparison of the proposed anomaly network with state-of-the-art frame-based anomaly networks in terms of AUC and Equal Error Rate (EER). Dataset details are as mentioned in the previous sections.

**Method**	**AUC (%)**	**EER (%)**
AnoDet A (Predicted image) (Schlegl et al., 2017)	86	11
AnoDet B (Chong and Tay, 2017)	59	43
AnoDet C (Hasan et al., 2016)	92	14
AnoDet D (Liu et al., 2018)	93	18
AnoDet E (Predicted feature) (Schlegl et al., 2017)	92%	15
AnoDet F (William et al., 2016)	89	15
Proposed (EvAn)	96	13

#### 4.5.2. Computational Complexity Analysis

The conventional frame-based anomaly networks are made up of deep generator networks. Moreover, they use an additional network such as optical flow or temporal autoencoder, or LSTM to capture the motion analytic. This additional network results in substantial computational complexity. In order to mitigate this effect, the proposed solution includes SSC layers (as mentioned in section 3) to build the generator of the proposed anomaly network and a thin encoder network (DL memory surface network) in the place of optical flow networks. These networks exploit the benefits of the sparsity of the event data and the time information encoded in the event data, respectively. These properties make our solution a desirable choice with good prediction accuracy and lesser computation (as shown in Table 7), more suitable for real-time hardware implementations with less computation budget.

**Table 7:** FLOPs (Floating Point OPerations) of conventional frame-based anomaly detection networks in comparison to the proposed network (128 × 128).

**Methods**	**FLOPs**
Ravanbakhsh et al. (2017a)	2.8 × 10^10^
Ravanbakhsh et al. (2017b)	2.8 × 10^10^
Liu et al. (2018)	6.4 × 10^10^
Proposed $\left(\frac{N_{a}}{mn}=0.25,N_{k}^{2}=1\right)$	**4.1 × 10^7^**
Proposed $\left(\frac{N_{a}}{mn}=0.0625,N_{k}^{2}=1\right)$	**8.6 × 10^6^**
Proposed $\left(\frac{N_{a}}{mn}=0.25,N_{k}^{2}=2\right)$	**8.3 × 10^7^**
Proposed $\left(\frac{N_{a}}{mn}=0.0625,N_{k}^{2}=2\right)$	**2.08 × 10^7^**
Proposed $\left(\frac{N_{a}}{mn}=0.0625,N_{k}^{2}=5\right)$	**1.04 × 10^8^**

*As the sparsity provided by the event camera is utilized optimally with Submanifold Sparse Convolution (SSC) layers, the proposed anomaly detection network displays a huge reduction in computational complexity. Proposed method experiment results are provided in bold*.

## 5. Conclusion

This paper presented the first baseline for the event-based sparse convolutional anomaly detection model. The proposed solution involves cGAN with SSC layers, which capture the anomaly in the event domain with reduced computational complexity. We have also proposed an unsupervised DL solution to effectively encode the time information encoded in the event data into a sparse DL memory surface. The sparsity of the DL memory surface could be controlled by tuning the sparsity term in the loss function. DL memory surface network allows us to replace computationally costly networks such as optical flow network, which is generally included in the design of vision tasks to capture motion features.

Furthermore, our DL memory surface is data dependant (unsupervised), unlike supervised DL-based event data representations proposed earlier in the literature. Unsupervised learning makes our solution a preferred choice for any motion-based video analytic task irrespective of the task at hand. We have also provided an event-based anomaly dataset on which the proposed algorithm has been validated from different perspectives. In addition to the empirical analysis of the proposed solution, we have also furnished analytical explanations of the proposed networks in Supplementary Material. It has also been displayed that the proposed method utilizes the advantages obtained from the event sensor effectively. The proposed solution resulted in considerable computation savings without compromising on performance compared to state-of-the-art conventional frame-based anomaly detection networks. Thus, making it a suitable solution for resource-constrained hardware platforms.

## Data Availability Statement

The original contributions presented in the study are included in the article/Supplementary Material, further inquiries can be directed to the corresponding author/s.

## Author Contributions

All authors listed have made a substantial, direct and intellectual contribution to the work, and approved it for publication.

## Conflict of Interest

The authors declare that the research was conducted in the absence of any commercial or financial relationships that could be construed as a potential conflict of interest.

## Publisher's Note

All claims expressed in this article are solely those of the authors and do not necessarily represent those of their affiliated organizations, or those of the publisher, the editors and the reviewers. Any product that may be evaluated in this article, or claim that may be made by its manufacturer, is not guaranteed or endorsed by the publisher.

## References

[B1] Alonso I. Murillo A. (2019). Ev-segnet: Semantic segmentation for event-based cameras, in IEEE Workshop on Computer Vision and Pattern Recognition (Long Beach, CA).

[B2] Annamalai L. Chakraborty A. Thakur C. S. (2019). Neuromorphic vision: from sensors to event based algorithms. WIREs Data Min. Knowl. Dis. 9:e1310. 10.1002/widm.1310

[B3] Brox T. Bruhn A. Papenberg N. Weickert J. (2004). High accuracy optical flow estimation based on a theory for warping, in European Conference on Computer Vision (Prague).

[B4] Calabrese T. Easthope A. (2019). Dhp19: dynamic vision sensor 3d human pose dataset, in IEEE Workshop on Computer Vision and Pattern Recognition (Long Beach, CA).

[B5] Chalapathy R. Menon A. K. Chawla S. (2017). Robust, deep and inductive anomaly detection, in European Conference on Machine Learning and Principles and Practice of Knowledge Discovery (Skopje).

[B6] Chen R. Li L. Delbruck T. Liu C. (2021). Reducing latency in a converted spiking video segmentation network, in IEEE International Symposium on Circuits and Systems (ISCAS) (Daegu).

[B7] Chong Y. S. Tay Y. H. (2017). Abnormal event detection in videos using spatiotemporal autoencoder, in International Symposium on Neural Networks (Sapporo), 189–196.

[B8] Choy Ch. Gwak J. Savarese S. (2019). 4d spatio-temporal convnets: minkowski convolutional neural networks, in Proceedings of the IEEE Conference on Computer Vision and Pattern Recognition (Long Beach, CA).

[B9] Ciccone M. Romanoni A. Matteucci M. (2020). A differentiable recurrent surface for asynchronous event-based data, in European Conference on Computer Vision.

[B10] Delbruck T. Barranco B. L. (2010). Activity-driven, event-based vision sensors, in Proceedings of IEEE International Symposium on Circuits and Systems (Sapporo).

[B11] Delbruck T. Mead C. (1989). An electronic photoreceptor sensitive to small changes in intensity, in NIPS. (Denver, CO).

[B12] Diederik M. W. Kingma P. (2014). Stochastic gradient vb and the variational auto-encoder, in Proceedings of the 2nd International Conference on Learning Representations (ICLR).

[B13] Gallego D. T. Orchard G. Bartolozzi C. Taba B. Censi A. (2019). Event-based vision: a survey. arXiv preprint arXiv:1904.08405. 10.1109/TPAMI.2020.3008413 32750812

[B14] Gehrig D. Loquercio A. Derpanis K. G. Scaramuzza D. (2019). End-to-end learning of representations for asynchronous event-based data, in Proceedings of the IEEE International Conference on Computer Vision (Seoul).

[B15] Goodfellow I. Pouget-Abadie J. (2014). Generative adversarial nets, in Advances in Neural Information Processing Systems (Montreal, QC), 2672–2680.

[B16] Graham B. (2014). Spatially-sparse convolutional neural networks. arXiv preprint arXiv:1409.6070.

[B17] Graham B. van der Maaten L. (2017). Submanifold sparse convolutional networks. arXiv preprint arXiv:1706.01307.

[B18] Hasan M. Choi J. Neumann J. Chowdhury R. Davis (2016). Learning temporal regularity in video sequences, in IEEE Conference on Computer Vision and Pattern Recognition (Las Vegas, NV), 733–742.

[B19] Hinton G. E. Salakhutdinov R. R. (2006). Reducing the dimensionality of data with neural networks. Science, 313(5786):504–507. 16873662 10.1126/science.1127647

[B20] Iandola F. N. Han S. Moskewicz M. W. Ashraf K. Dally W. J. Keutzer K. (2016). Squeezenet: alexnet-level accuracy with 50x fewer parameters and 1mb model size. arXiv preprint arXiv:1602.07360.

[B21] Isola Y. Zhu J.-Y Zhou T. Efros A. A. (2017). Image-to-image translation with conditional adversarial networks, in IEEE Conference on Computer Vision and Pattern Recognition (Honolulu, HI), 1125–1134.

[B22] Joo S. Chellappa R. (2006). Attribute grammar-based event recognition and anomaly detection, IEEE Workshop on Computer Vision and Pattern Recognition (New York, NY), 107–107.

[B23] Kiran B. R. Thomas D. Parakkal R. (2018). An overview of deep learning based methods for unsupervised and semi-supervised anomaly detection in videos. J. Imaging 4:36. 10.3390/jimaging4020036

[B24] Lagorce X. Orchard G. Galluppi F. Shi B. E. Benosman R. B. (2017). Hots: a hierarchy of event-based time-surfaces for pattern recognition. IEEE Trans. Pattern Anal. Mach. Intel. 39, 1346–1359. 10.1109/TPAMI.2016.2574707 27411216

[B25] Li H. Liu H. Ji X. Li G. Shi L. (2017). Cifar10-dvs: an event-stream dataset for object classification. Front. in Neurosci. 11:309. 10.3389/fnins.2017.00309 28611582 PMC5447775

[B26] Lichtsteiner P. Posch C. Delbruck T. (2008). A 128 x 128 120 db 15 us latency asynchronous temporal contrast vision sensor. IEEE J. Solid State Circ. 43, 566–576. 10.1109/JSSC.2007.914337

[B27] Lin M. Chen Q. Yan S. (2013). Network in network. arXiv preprint arXiv:1312.4400.

[B28] Liu W. Luo W. Lian D. Gao S. (2018). Future frame prediction for anomaly detection–a new baseline, in IEEE Conference on Computer Vision and Pattern Recognition (Salt Lake City, UT), 6536–6545.

[B29] Luo W. Liu W. Gao S. (2017). Remembering history with convolutional lstm for anomaly detection, in IEEE International Conference on Multimedia and Expo (ICME), 439–444.

[B30] Maqueda A. I. Loquercio A. Gallego G. García N. Scaramuzza D. (2018). Event-based vision meets deep learning on steering prediction for self-driving cars, in IEEE Conference on Computer Vision and Pattern Recognition (Salt Lake City, UT), 5419–5427.

[B31] Medel J. R. (2016). Anomaly detection using predictive convolutional long short-term memory units, in Rochester Institute of Technology (Rochester, NY).

[B32] Medel J. R. Savakis A. (2016). Anomaly detection in video using predictive convolutional long short-term memory networks. arXiv preprint arXiv:1612.00390.

[B33] Miguel J. C. S. Martinez J. M. (2008). Robust unattended and stolen object detection by fusing simple algorithms, in Advanced Video and Signal Based Surveillance (Taipei), 18–25.

[B34] Mitrokhin Y. Mitrokhin A. Parameshwara C. Fermuller C. Yorke J. A. Aloimonos Y. (2019). Unsupervised learning of dense optical flow and depth from sparse event data. arXiv:1809.08625.

[B35] Moeys P. D. Corradi F. Li C. Bamford S. Longinotti L. Voigt F.. (2017). A sensitive dynamic and active pixel vision sensor for color or neural imaging applications. IEEE Trans. Biomed. Circ. Syst. 12, 123–136. 10.1109/TBCAS.2017.2759783 29377801

[B36] Mueggler E. Rebecq H. Gallego G. Delbruck T. Scaramuzza D. (2017). The event-camera dataset and simulator: Event-based data for pose estimation, visual odometry, and slam. Int. J. Robot. Res. 36, 142–149. 10.1177/0278364917691115

[B37] Ng A. (2011). Sparse autoencoder. CS294A Lect. Notes 72, 1–19.

[B38] Nguyen A. Do T. Caldwell D. G. Tsagarakis N. G. (2017). Real-time pose estimation for event cameras with stacked spatial lstm networks. arXiv preprint arXiv:1708.09011.

[B39] Park P. Cho B. H. (2016). Performance improvement of deep learning based gesture recognition using spatiotemporal demosaicing technique, in Image Processing (ICIP), 2016 IEEE International Conference on Vol. 4, (Phoenix, AZ,), 31624–1628.

[B40] Posch C. Matolin D. Wohlgenannt R. (2008). An asynchronous timebased image sensor, in IEEE International Symposium on Circuits and Systems (Seattle, WA), 2130–2133.

[B41] Ravanbakhsh M. Nabi M. Sangineto E. Marcenaro l. Regazzoni C. Sebe N. (2017a). Abnormal event detection in videos using generative adversarial nets, in IEEE International Conference on Image Processing (ICIP) (Beijing), 1577–1581.

[B42] Ravanbakhsh M. Sangineto E. Nabi M. Sebe N. (2017b). Training adversarial discriminators for cross-channel abnormal event detection in crowds. CoRR, vol. abs/1706.07680.

[B43] Robey D. E. Thio W. Iu H. C. C. Eshraghian J. k. (2021). Naturalizing neuromorphic vision event streams using generative adversarial networks, in IEEE International Symposium on Circuits and Systems (ISCAS) (Daegu).

[B44] Rueckauer B. Lungu I.-A. Hu Y. Pfeiffer M. Liu S.-C. (2017). Conversion of continuous-valued deep networks to efficient event-driven networks for image classification. Front. Neurosci. 11:682. 10.3389/fnins.2017.00682 29375284 PMC5770641

[B45] Russell A. Orchard G. Dong Y. Mihalas S. Niebur E. Tapson J. Cummings R. E. (2010). Optimization methods for spiking neurons and networks. IEEE Trans. Neural Netw. 21, 123–136. 10.1109/TNN.2010.2083685 20959265 PMC3164281

[B46] Sabokrou M. Fathy M. Hoseini M. (2016). Video anomaly detection and localisation based on the sparsity and reconstruction error of auto-encoder. Electr. Lett. 52, 1122–1124.

[B47] Schlegl T. Seebock P. Waldstein S. M. Schmidt-Erfurth U. Langs G. (2017). Unsupervised anomaly detection with generative adversarial networks to guide marker discovery, in International Conference on Information Processing in Medical Imaging (Boone, NC), 146–157.

[B48] Serrano-Gotarredona T. Linares-Barranco B. (2013). A 128 x128 1.5% contrast sensitivity 0.9% fpn 3 μs latency 4 mw asynchronous frame-free dynamic vision sensor using transimpedance preamplifiers. IEEE J. Solid State Circ. 48, 827–838. 10.1109/JSSC.2012.2230553

[B49] Shu M. Guang C. Xiangyu N. Yang Z. Kejia R. Zhenshan B.. (2019). Neuromorphic benchmark datasets for pedestrian detection, action recognition, and fall detection. Front. Neurorobot. 13:38. 10.3389/fnbot.2019.00038 31275128 PMC6591450

[B50] Sironi A. Brambilla M. Bourdis N. Lagorce X. Benosman R. (2018). Hats: histograms of averaged time surfaces for robust event-based object classification, in IEEE Conference on Computer Vision and Pattern Recognition (Salt Lake City, UT), 1731–1740.

[B51] Szegedy C. Liu W. Jia Y. (2015). Going deeper with convolutions, in 2015 IEEE Conference on Computer Vision and Pattern Recognition (CVPR) (Boston, MA).

[B52] Thakur C. S. Molin J. L. Cauwenberghs G. Indiveri G. Kumar K. Qiao N.. (2018). Large-scale neuromorphic spiking array processors: a quest to mimic the brain. Front. Neurosci. 12:891. 10.3389/fnins.2018.00891 30559644 PMC6287454

[B53] Wang L. Ho Y. S. Yoon K. J. (2019). Event-based high dynamic range image and very high frame rate video generation using conditional generative adversarial networks, in IEEE Conference on Computer Vision and Pattern Recognition (Long Beach, CA).

[B54] William L. Gabriel K. David C. (2016). Deep predictive coding networks for video prediction and unsupervised learning. arXiv preprint arXiv:1605.08104.

[B55] Yan M. Jiang X. Yuan J. (2018). 3d convolutional generative adversarial networks for detecting temporal irregularities in videos, in 2018 24th International Conference on Pattern Recognition (ICPR) (Beijing).

[B56] Zhu A. Yuan L. Chaney K. Daniilidis K. (2018). Ev-flownet: self-supervised optical flow estimation for event-based cameras, in Proceedings of Robotics: Science and Systems (Pittsburgh, PA).

[B57] Zhu A. Z. Yuan L. Chaney K. Daniilidis K. (2019). Unsupervised event-based learning of optical flow, depth, and egomotion, in IEEE Coference on Computer Vision and Pattern Recognition, 989–997.

