# Supplementary material for: EvAn: Neuromorphic Event-Based Sparse Anomaly Detection
Source: Front Neurosci. 2021 Jul 29;15:699003. doi: 10.3389/fnins.2021.699003 (PMC8358807; doi:10.3389/fnins.2021.699003)
Supplement: Supplementary file 1 [file Data_Sheet_1.PDF]

Supplementary of the paper EvAn: Neuromorphic Event-based Sparse Anomaly Detection

Lakshmi Annamalai, Anirban Chakraborty, Member, IEEE, and Chetan Singh Thakur, Senior Member, IEEE.

1 Introduction

In Sec. 1.1 and 1.2, we describe our implementation and different metrics we have used to assess the proposed solution. In Sec. 1.3, we provide additional empirical validation for our dataset's complexity. In Sec. 1.4, we present the results of the experiments conducted to tackle the noise effects of event camera. Section 1.5 analyses the performance of DL memory surface network on two class activity recognition task in terms of t-SNE plot. Section 1.6 presents a visual display of a sample of state-of-the-art event representations with which our DL memory surface has been compared with. Sec. 1.7 provides experimental results to have insight into the latency introduced by the proposed deep learning based event representation solution compared to that of non-deep learning conventional solutions. Sec 1.8 and Sec 1.9 furnishes the results of self-analysis of anomaly detection network in terms of classification performance and computational complexity. Sec 1.10 provides the details of the conventional frame-based state-of-the-art anomaly networks considered in this paper for comparative analysis.

1.1 Implementation Details

The input discretized event volume, formed by accumulating events as described in section 3 of the paper, is passed through a $3D$ convolution layer, which has a receptive field of $1 \times 1 \times C$, where C is the number of channels. DL memory surface network, initialized with random weights sampled from a standard normal distribution, is trained with these input event volume to reconstruct its inputs. The activation regularization of the DL memory surface network is fixed for anomaly detection network experiments. We extract the DL memory surface from the bottleneck layer of the DL memory surface network. We used $2e-4$ learning rate and 0.5 momentum.

Sparse convolutional cGAN anomaly detection network is trained with the DL memory surface of normal events to predict future normal events. The generator's architecture is a sparse convolutional encoder-decoder, and that of the discriminator is a classifier architecture, the details of which are provided in section 3 of the paper. DL memory surface is fed to the generator network as a sparse tensor whose value is defined only at discrete locations specified by the indices matrix. Sparse tensor representation is crucial to saving memory space and maintaining sparsity in the generator network's computation. The optimization technique used is Stochastic Gradient Descent (SGD) with a learning rate of 0.1 and momentum of 0.9.

1.2 Evaluation Metrics

The various criteria used to evaluate the DL memory surface network and anomaly detection network are recall, precision, F1 score, and accuracy. All these measures are based on true-positive rates (TPR), false-positive Rates (FPR), true-negative rates (TNR), and false-negative rates (FNR), where positive and negative denote the presence and absence of negative events.

The recall is the classifier's ability to recall positive classes (Eq. 1). Precision (Eq. 1) is the ratio between the true-positive rate and the total number of retrieved images. F1-measure (Eq. 1) indicates the balance between precision and recall. Accuracy (Eq. 1) is the fraction of true predictions of the model. In addition to this, the Equal Error Rate (EER) is also used to summarize the anomaly detection network's performance. EER is the ratio of frames that are misclassified at $FPR = 1 - TPR$.

$$
\begin{aligned}
Recall &= \frac{TPR}{TPR+FNR} \\
Precision &= \frac{TPR}{TPR+FPR} \\
F1 &= \frac{2*precision*recall}{precision+recall} \\
Accuracy &= \frac{TPR+TNR}{TPR+TNR+FPR+FNR}
\end{aligned}
\tag{1}
$$

- *Lakshmi Annamalai is with Defence Research and Development Organization, Bangalore, India. This work was done at NeuRonICS lab, Department of Electronic Systems Engineering, Indian Institute of Science, Bangalore, India, where Lakshmi is currently pursuing her doctoral research*
  E-mail: lakshmia@iisc.ac.in
- *Anirban Chakraborty is with Department of Computational and Data Sciences at Indian Institute of Science, Bangalore, India.*
  E-mail: anirban@iisc.ac.in
- *Chetan Singh Thakur is with Department of Electronic Systems Engineering at Indian Institute of Science, Bangalore, India.*
  E-mail: csthakur@iisc.ac.in

1.3 Dataset: Event Rate

To emphasize the complexity of the dataset considered, we have estimated the event rates of various anomalous activities vs. normal activity at various time instants for a uniform time interval. Fig. 2 shows the histogram of event rates (over a period of 10 ms) for normal and anomalous activity, and Fig. 1 displays the number of events (over a time period of 50 ms) vs. various time instants for normal and anomalous activities. It could be seen that there is a lot of overlap between normal and anomalous activities in terms of event rate, which indicates that the rate of motion of the normal and anomalous activities resemble each other.

1.4 DL Memory Surface Network: Input

Fig. 3 shows discretized event slices for various ΔT of $10ms, 30ms, 50ms$. As the event camera data has noise effects, we have done preprocessing to remove the same. It could be visualized that no information is retained after noise removal when $\Delta T = 10ms, 30ms$. Hence we have fixed the accumulation time of events to $50ms$ to have an optimum trade-off between temporal latency and information content.

1.5 DL Memory Surface Network: t-SNE Analysis on Two Class Activity Recognition

These experiments evaluate the DL memory surface network's benefits quantitatively in encoding information into bottleneck layer features. To prove the proposed DL memory surface network's generalization, we have evaluated it on a completely different vision task known as activity recognition.

The DL memory surface network was trained on discretized event volumes. Subsequent to freezing the model, DL memory surfaces are extracted from the network's bottleneck layer for different activities and subjected to feature extraction with MobileNet. The extracted features are analyzed in terms of two class activity recognition task (normal vs. anomaly). The number of samples is carefully chosen to balance the two classes.

For the purpose of visualization, the dimension of the features is reduced to two using t-SNE. Fig. 4 shows the $2D$ t-SNE embedding of MobileNet features of various anomalous activities vs. normal activity. Better clustering of DL memory surface features is evident, proving that the network has learned the information which was encoded in the events.

1.6 DL Memory Surface Network: Conventional Representations of Event Data

Fig. 5 gives the visualization of different state-of-the-art conventional hand-crafted event representations (EvSAE, EvSNN, EvFreq, EvFlow, EvCount). The references and comparison with the proposed approach are provided in the paper.

Number of Events	SAE	SNN	Freq	EvOn	EvOff	DLmem
49806	0.008	22.49	0.011	0.025	0.020	0.055
54698	0.008	20.77	0.010	0.023	0.019	0.055
52387	0.008	18.71	0.013	0.030	0.023	0.055
50530	0.008	16.6	0.010	0.022	0.018	0.055
62310	0.011	14.9	0.013	0.028	0.024	0.055

TABLE 1: Comparison of run time (in ms) of the proposed DL memory surface network with other hand crafted methods whose details are provided in the paper.

Anomaly	AUC	EER	Avg Precision	F1 Score
**Falling**	96%	14%	94%	83%
**GetUp**	93%	18%	88%	75%
**Jumping**	99%	1%	99%	80%
**Kicking**	83%	24%	82%	56%
**Picking**	82%	23%	78%	72%
**Sit**	82%	25%	74%	87%

TABLE 2: Quantitative analysis of proposed anomaly detection network in terms of AUC, EER, average precision, and F1 score

1.7 DL Memory Surface Network: Computational Time Analysis

An event camera's main advantage is low latency, which could be retained while processing if it involves asynchronous processing. However, it results in reduced accuracy. To trade latency for accuracy, researchers have proposed to accumulate events over a period of time. The proposed approach is one such approach where we have used a deep learning solution to learn representation from the data. As we may be actually sacrificing speed for improved accuracy, we also provide the computational complexity of the proposed approach with respect to non-deep learning approaches for a different number of input events. Table. 1 furnishes the computation time vs. the number of events, which is performed on a CPU (Intel i7 CPU, 64bits, 2.3GHz and 64 GB of RAM) with GPU (GeForce RTX 1080 Ti). It can be seen from Table. 1 that the proposed approach is still a real-time solution because of the thin encoder network that we have proposed for DL memory surface generation.

1.8 Anomaly Detection Network: Quantitative Self Analysis

In this section, we quantitatively assess the performance of the proposed system as a whole. This is an analysis of the network's capability to predict abnormal pixels' presence, based on which abnormal label is given to that particular set of events. Abnormality detection is evaluated over a range of thresholds to construct different metrics such as AUC, EER, average precision, and F1 score, etc., on different anomalies. Quantitative analysis in terms of AUC, EER, average precision, and F1 score is furnished in Table. 2. It could be visualized that the proposed method performs well even for anomalies that do not differ from normal activities in terms of rate of motion (or events generated) such as sitting, picking, and kicking. Table. 3 displays a detailed analysis of FPR and precision at different TPR / recall on a sub-set of proposed anomalies.

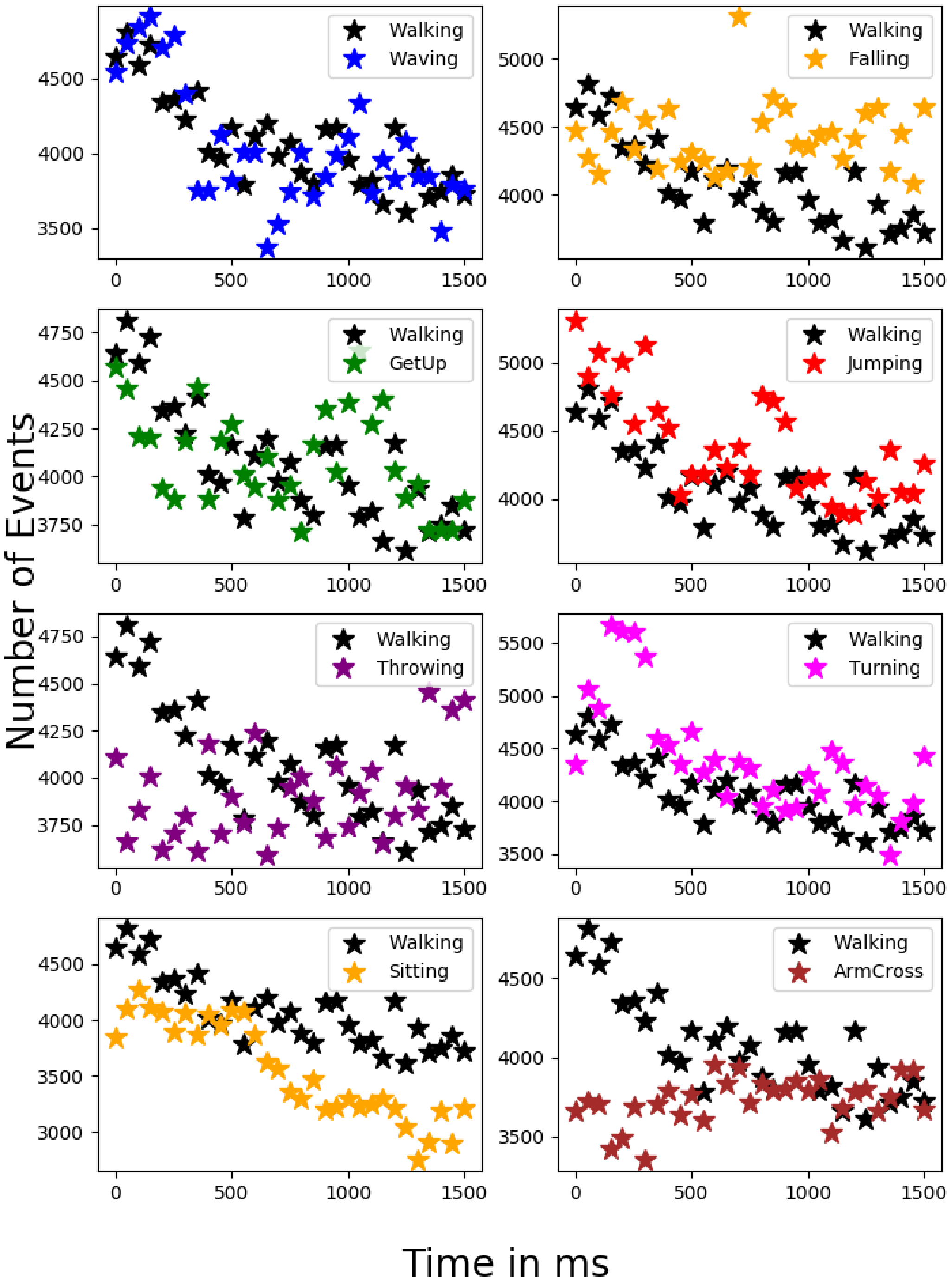

Fig. 1: Display of event rate of normal vs. anomalous activity at differnt time instants for standard time interval of 50 ms. Huge overlap in the event rate indicates that the anomalous activities considered does not differ from normal activity in terms of rate of motion.

Falling			GetUp			Kicking			Picking			Sitting		
TPR/R	FPR	Prec	TPR/R	FPR	Prec	TPR/R	FPR	Prec	TPR/R	FPR	Prec	TPR/R	FPR	Prec
1.0	1.0	0.33	1.0	1.0	0.28	1.0	1.0	0.29	1.0	1.0	0.47	1.0	1.0	0.46
0.92	0.29	0.60	0.95	0.4	0.48	0.8	0.56	0.37	0.98	0.79	0.52	0.98	0.9	0.49
0.89	0.18	0.70	0.77	0.10	0.73	0.76	0.16	0.65	0.93	0.31	0.72	0.8	0.4	0.68
0.86	0.07	0.84	0.72	0.05	0.84	0.64	0.02	0.94	0.28	0.05	0.8	0.50	0.26	0.73
0.78	0.02	0.93	0.68	0.0	1.0	0.48	0.0	1.0	0.2	0.0	1.0	0.14	0.02	0.85

TABLE 3: Quantitative analysis of anomaly network in terms of recall (R), precision (Prec), TPR and FPR for various anomalous activities.

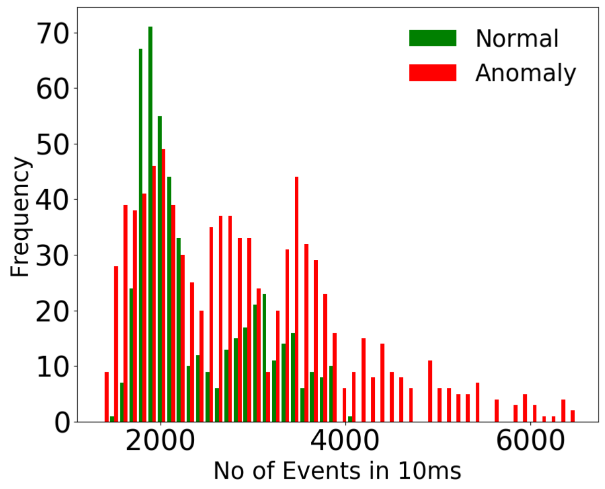

Fig. 2: Display of histogram of event rate of normal vs anomalous activity. The event rates are estimated for a standard time interval of 10 ms. Huge overlap in the histogram of event rate indicates that the anomalous activities considered does not differ from normal activity in terms of rate of motion.

1.9 Anomaly Detection Network: Computation Analysis

Table. 4 gives the comparison of dense convolution and Submanifold Sparse Convolution (SSC) layers at different levels in terms of FLOPs. The terms inside the bracket provide the ratio between the FLOPs of the corresponding dense layer and the SSC layer. The higher the value, the better the save in computation achieved with SSC layers. We have provided the SSC layer's computational complexity at two different N_k^2 (notations explained in the main paper) and two different sparsity levels. We have assumed N_k to be constant over the given image/feature map. Compared to dense layers, SSC layers requires far lesser computation, especially when $\left(\frac{mn}{N_a} = 0.0625, N_k^2 = 1\right)$.

1.10 Anomaly Detection Network: Details of Conventional Frame-based Anomaly Networks used for Comparison

AnoDet B (reference given in section 4.3.2 of paper) is a Spatio-temporal auto-encoder architecture, which is made up of spatial and temporal autoencoders to learn spatial features and temporal patterns, respectively. The discretized volume of events mentioned in section 3 of the paper is fed as input to the architecture. The event frames are classified as normal and anomalous based on the reconstruction error of input event volume. We have adapted the code implementation provided at https://github.com/harshtikuu/Abnormal_Event_Detection

AnoDet C (reference given in section 4.3.2 of paper) uses a fully convolutional autoencoder in addition to conventional motion feature descriptors to learn low and high-level features. The input is constructed as T event frames of discretized event volume stacked together. The parameters are fixed as the implementation provided at https://github.com/NRauschmayr/Anomaly_Detection. The reconstruction error is estimated as the sum of per pixel error, based on which an event frame is classified as normal or anomaly.

AnoDet F (reference given in section 4.3.2 of paper) is a deep recurrent convolutional neural network for future frame prediction. The implementation we have used is the architecture implemented as a custom layer in Keras https://github.com/coxlab/prednet. To predict future frame accurately, it needs a sequence of event frames of discretized event volume as input to learn the objects' motion dynamics. An event frame is classified as normal or anomaly based on the prediction error.

AnoDet E and AnoDet A (reference given in section 4.3.2 of paper) are the two versions of an anomaly detection network, which utilizes predicted feature and image respectively for anomaly detection. The architecture is made up of deep convolutional GAN, and it works on static images. The input is individual event frames formed by discretizing the events. It provides an anomaly score as a measure of fit of the event frame under consideration to that of the normal image model that it has learned during training. The TensorFlow implementation is available at https://github.com/tSchlegl/f-AnoGAN.

AnoDet D (reference given in section 4.3.2 of paper) depends on the optical flow network to extract motion information for anomaly detection. Hence, it has been tested without training, which leads to deficient performance. Implementation is available at https://github.com/StevenLiuWen/ano_pred_cvpr2018

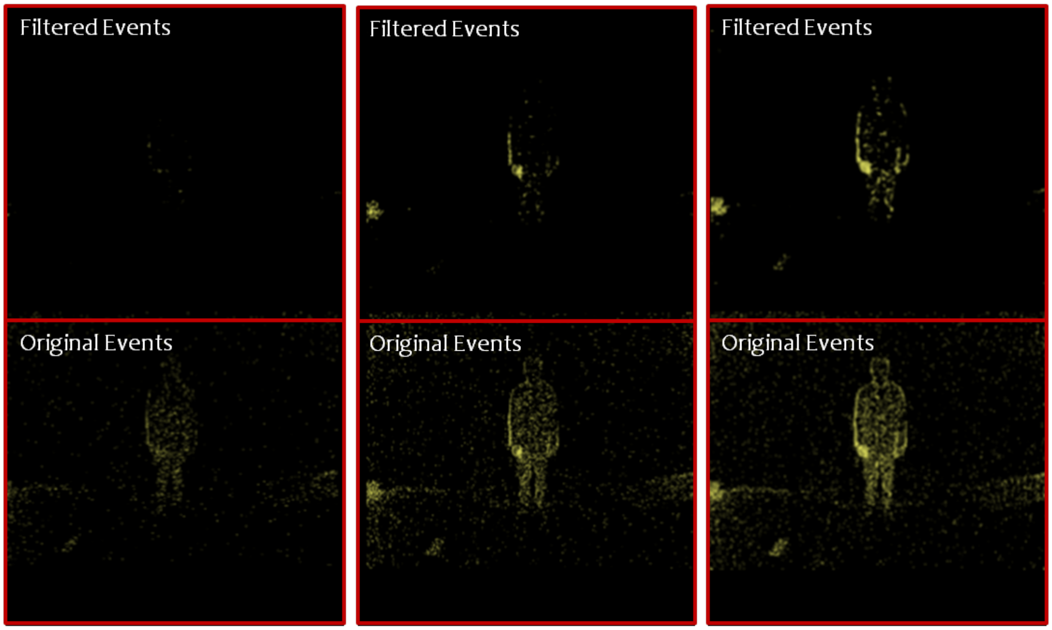

(a) $10ms$ (b) $30ms$ (c) $50ms$

Fig. 3: Visualization of discretized events (bottom) and noise filtered events (top) accumulated over different time periods of $10ms$ (left), $30ms$ (middle) and $50ms$ (right)

Layer	Cin	Cout	m,n	D	S1	S2	S3	S4	S5
Layer 1	1	16	256, 256	6684672	262144	65536	786432	196608	2359296
Layer 2	16	32	128, 128	150470656	4063232	1015808	8257536	2064384	20840448
Layer 3	32	64	64, 64	150732800	4128768	1032192	8323072	2080768	20905984
Layer 4	64	128	32, 32	150863872	4161536	1040384	8355840	2088960	20938752
Layer 5	128	256	16, 16	150929408	4177920	1044480	8372224	2093056	20955136
Layer 6	256	512	8, 8	150962176	4186112	1046528	8380416	2095104	20963328

TABLE 4: Comparison of FLOPs of dense (D) and SSC layers S1 $\left(\frac{N_a}{mn} = 0.25, N_k^2 = 1\right)$, S2 $\left(\frac{N_a}{mn} = 0.0625, N_k^2 = 1\right)$, S3 $\left(\frac{N_a}{mn} = 0.25, N_k^2 = 2\right)$, S4 $\left(\frac{N_a}{mn} = 0.0625, N_k^2 = 2\right)$, S5 $\left(\frac{N_a}{mn} = 0.25, N_k^2 = 5\right)$ at various layers. Maximum benefit in terms of computation is achieved when $\left(\frac{mn}{N_a} = 0.0625, N_k^2 = 1\right)$

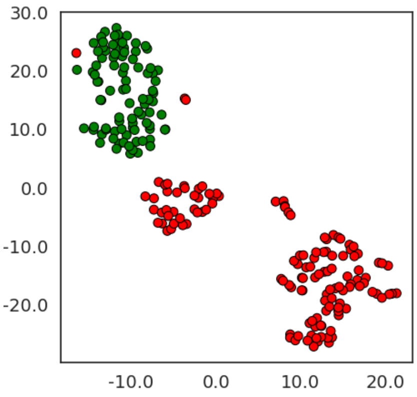

(a) Walking (red) vs. Waving (green)

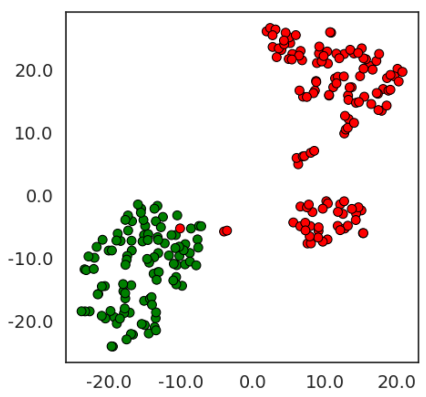

(b) Walking (red) vs. GetUp (green)

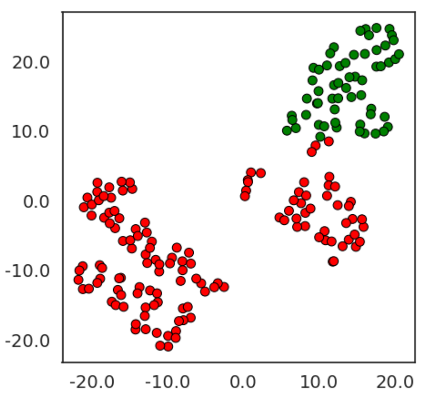

(c) Walking (red) vs. Throwing (green)

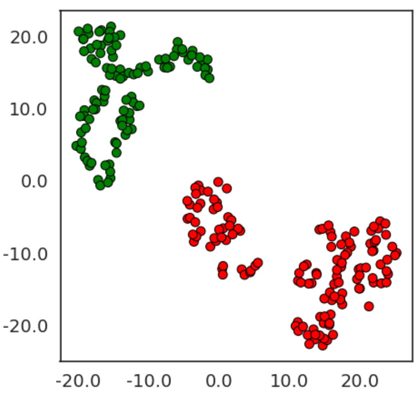

(d) Walking (red) vs. Jumping (green)

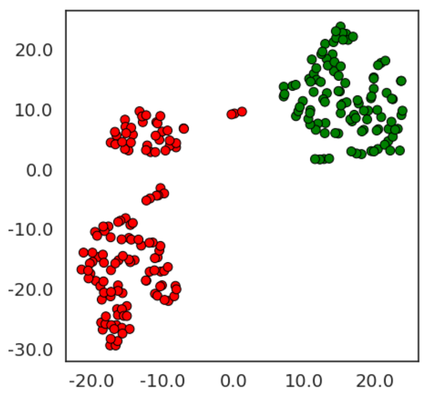

(e) Walking (red) vs. Sitting (green)

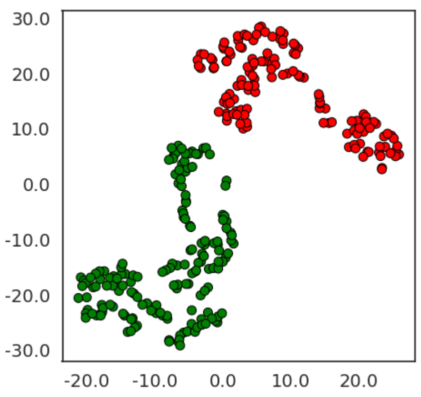

(f) Walking (red) vs. ArmCross (green)

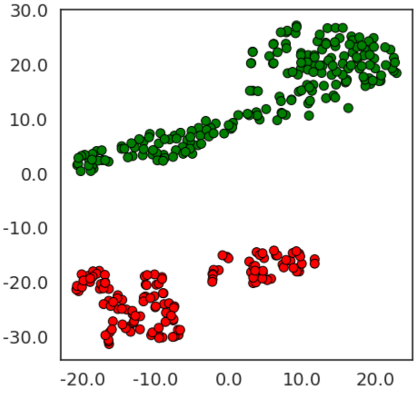

(g) Walking (red) vs. Falling (green)

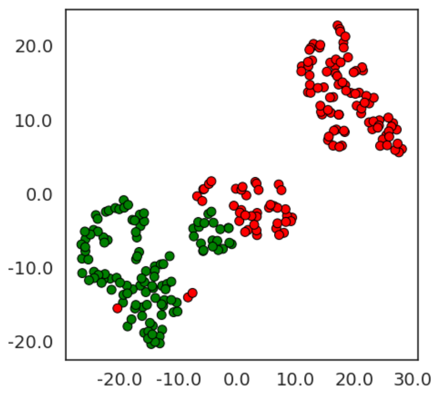

(h) Walking (red) vs. Tying (green)

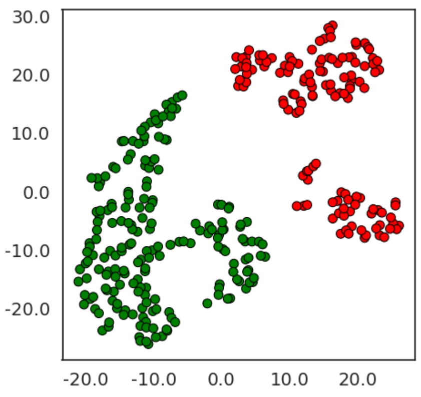

(i) Walking (red) vs. Kicking (green)

Fig. 4: MobileNet features are extracted for DL memory surface. Dimensionality of the features has been reduced to two using t-SNE. The projected two dimensional t-SNE features of various anomalous activities vs. normal activity (walking) are plotted. x and y axis are t-SNE features along dimension 1 and 2 respectively.

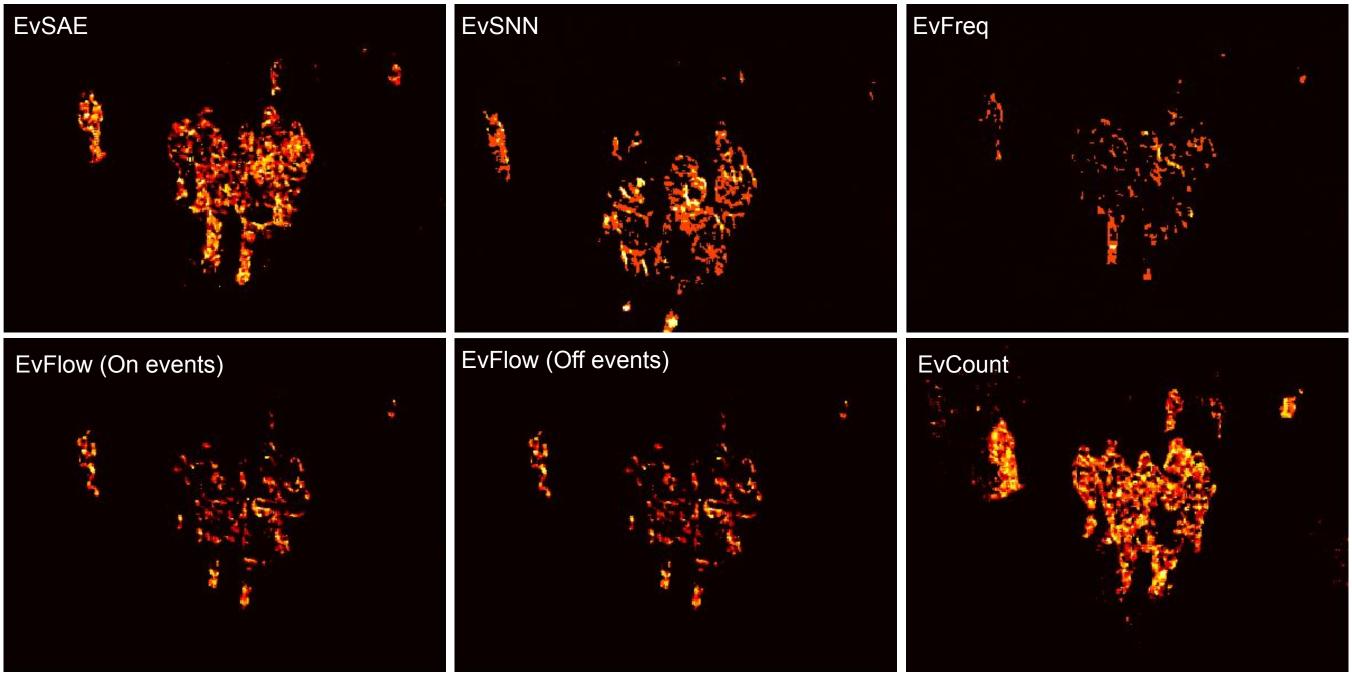

Fig. 5: Visualization of different event data representations proposed in literature (reference given in paper)
