# Supplementary material for: EvAn: Neuromorphic Event-Based Sparse Anomaly Detection
Source: Front Neurosci. 2021 Jul 29;15:699003. doi: 10.3389/fnins.2021.699003 (PMC8358807; doi:10.3389/fnins.2021.699003)
Supplement: Supplementary file 2 [file Data_Sheet_2.PDF]

Supplementary

In this supplementary, we perform a comprehensive assessment of our proposed DL memory surface generation network on event data. Experiments have been carried out for self-analysis and a thorough comparison with the existing state-of-the-art methods. In addition to this, we have also provided a detailed analytical explanation of the working of the networks proposed.

To substantiate the generality of our network, we have run all these experiments via a different vision task known as activity recognition. The complexity level of recognizing the actions is a challenging task as it involves understanding temporal context and spatial information. This mandates better learning of spatial and temporal information. The following experiments on DL memory surface as applied to activity recognition showcases our network's flexibility to be readily extended to any vision task. We note that our network exploits no domain knowledge of vision tasks.

1 Self Analysis

In this segment, we present the results of self-analysis of our proposed DL memory surface generation network, which enables us, i) To argue that the representation learned by the DL memory surface can perform better than the discretized event input volume, and ii) To validate the usage of sparsity in the DL memory surface network. Towards this, the DL memory surface network was trained on discretized event volumes of the temporal classes present in our dataset. Then, driven by the experiments conducted to mitigate the inherent noisy nature of event data, the discretized event volumes are formed by accumulating events over a ΔT of $50ms$. Subsequent to freezing the model, DL memory surfaces are extracted from the network's bottleneck layer for different activities.

1.1 Qualitative Analysis

This section provides a qualitative analysis of the DL memory surface. Towards visual analysis, we provide the display of the DL memory surface network's input and output for two different actions, bending and running (Fig. 1). The DL memory surfaces are subjected to feature extraction with MobileNet CNN feature extraction architecture [4]. For visualization, the dimension of the features is reduced to two using t-Distributed Stochastic Neighbor Embedding (t-SNE). Fig. 2 shows the t-SNE plot of DL memory surface features.

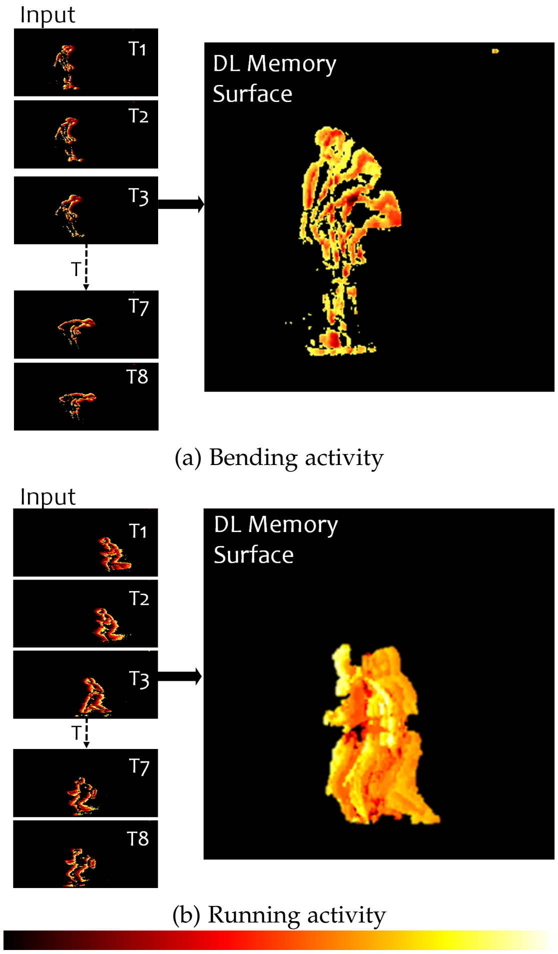

(a) Bending activity

(b) Running activity

(c) Colormap

Fig. 1: Visualization of discretized volumes of events (left) and DL memory surface (Right). It could be visualized that the DL memory surface captures the information regarding the history of event data. The events and DL memory surface are color-coded for better visualization. (c) Colormap (0: Black, 255: Yellow)

1.2 Comparison with Input Event Volume on Activity Recognition

These experiments evaluate the DL memory surface network's benefits quantitatively in encoding information into bottleneck layer features over the input event volume. To prove the proposed DL memory surface network's generalization, we have evaluated it on a completely different vision task known as activity recognition.

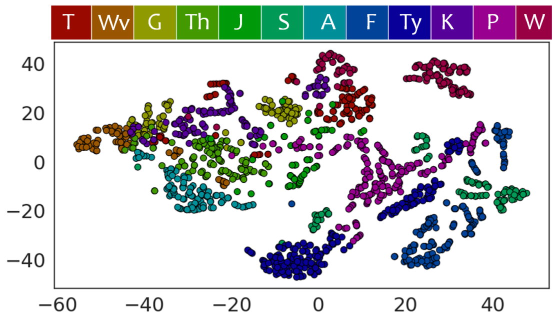

Fig. 2: MobileNet features are extracted for the DL memory surface. The dimensionality of the features has been reduced to two using t-SNE. The projected two-dimensional t-SNE features of various activities are plotted. x and y axes represent two dimensional projection of MobileNet [4] features. Color Legend: (T) Turning, (Wv) Waving, (G) GetUp, (Th) Throw, (J) Jumping, (S) Sit, (A) ArmCross, (F) Falling, (Ty) Tying, (K) Kicking, (P) Picking, (W) Walking.

	In	MS		In	MS
**GetUp**	0.81	**0.96**	**Turning**	0.79	0.81
**Jumping**	0.76	**0.91**	**Waving**	0.96	0.99
**Sit**	0.85	**0.92**	**Throwing**	0.95	0.97
**Tying**	0.81	**0.92**	**ArmCross**	0.97	0.99
**Kicking**	0.83	**0.92**	**Falling**	0.85	0.89
**Picking**	0.77	**0.88**			

TABLE 1: MobileNet features [4] are extracted for In and MS (DL memory surface). The features of various activities vs. walking are clustered using the K-Means clustering algorithm, and clustering accuracy is furnished. The clustering accuracy of the DL memory surface is greater than that of input event volume (especially on those activities given in column 1).

Input event volume and DL memory surface are subjected to feature extraction with MobileNet [4]. The extracted features are analyzed using the K-Means algorithm to recognize two-class activity recognition tasks (walking vs. other activity). The number of samples is carefully chosen to balance the two classes. Table. 1 shows the clustering accuracy of walking vs. various other activities. Given the higher clustering accuracy of the DL memory surface than raw event volume (especially on activities given in column 1), we prove that the proposed DL memory surface network is a valuable framework for any vision task.

1.3 Analysis for Sparsity on Activity Recognition

By placing limitations in the activation of the bottleneck layer in the DL memory surface network, we maintained the sparsity of the input events, which is essential to the application of sparse submanifold convolution in the cGAN anomaly detection network. It has been observed that exciting structures can be discovered by imposing sparsity constraints on the network's activations. In the DL memory surface network, we could tune the sparsity level to the desired level.

To compare the quality of features learned by the network at different sparsity levels, we have extracted the DL memory surface with many choices of activation regularizations. These sparse DL memory surfaces are tested for the general vision application, which is activity recognition. Towards this, these sparse DL memory surfaces are subjected to MobileNet feature extraction. The usefulness of the features is evaluated by exploring the classification capability of an SVM classifier on activities present in our dataset. Fig. 3 shows the plots of different metrics such as accuracy, recall, precision, and F1 measures of activity recognition for various sparsity levels averaged across different activities. The sparsity ratio is estimated as the ratio between the sparsity of the output DL memory surface and the input events. The DL memory surface generated with a sparsity ratio of 0.62 performs well in various classification metrics.

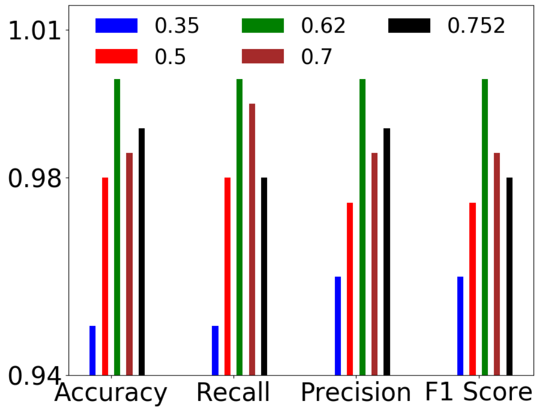

Fig. 3: MobileNet features are extracted for DL memory surface generated with a wide range of sparsity ratios. Activity recognition was tried on these features with SVM as a classifier. The plot shows the classification metrics vs. different sparsity ratio, optimum accuracy being achieved at a sparsity ratio of 0.62

2 Performance Comparison with State-of-the-Art

This section quantitatively assesses the performance of the proposed DL memory surface compared to existing memory surfaces. This is an interesting evaluation as this proves how well our DL memory surface network learns the information from the data in contrast to the conventional fixed encoding of event memory surfaces. Furthermore, to confirm the effectiveness of the information captured by the proposed network irrespective of the vision task at hand, we conducted comparison experiments on the activity recognition task. As we have used a vision task different from anomaly detection, it proves that the proposed representation is data dependant, generic to any task, whereas [3] [1] is tailored to the task at hand.

The different event representations (visualization provided in Fig. 4) that have been used for comparison are Surface of Active Events (SAE) [5], Spiking Neural Network (SNN) model based on Leaky Integrate-and-Fire neuron model [5], frequency-based (Freq) [5], EvFlow [2]. Fig. 4 gives the visualization of different state-of-the-art conventional hand-crafted event representations (EvSAE, EvSNN, EvFreq, EvFlow, EvCount).

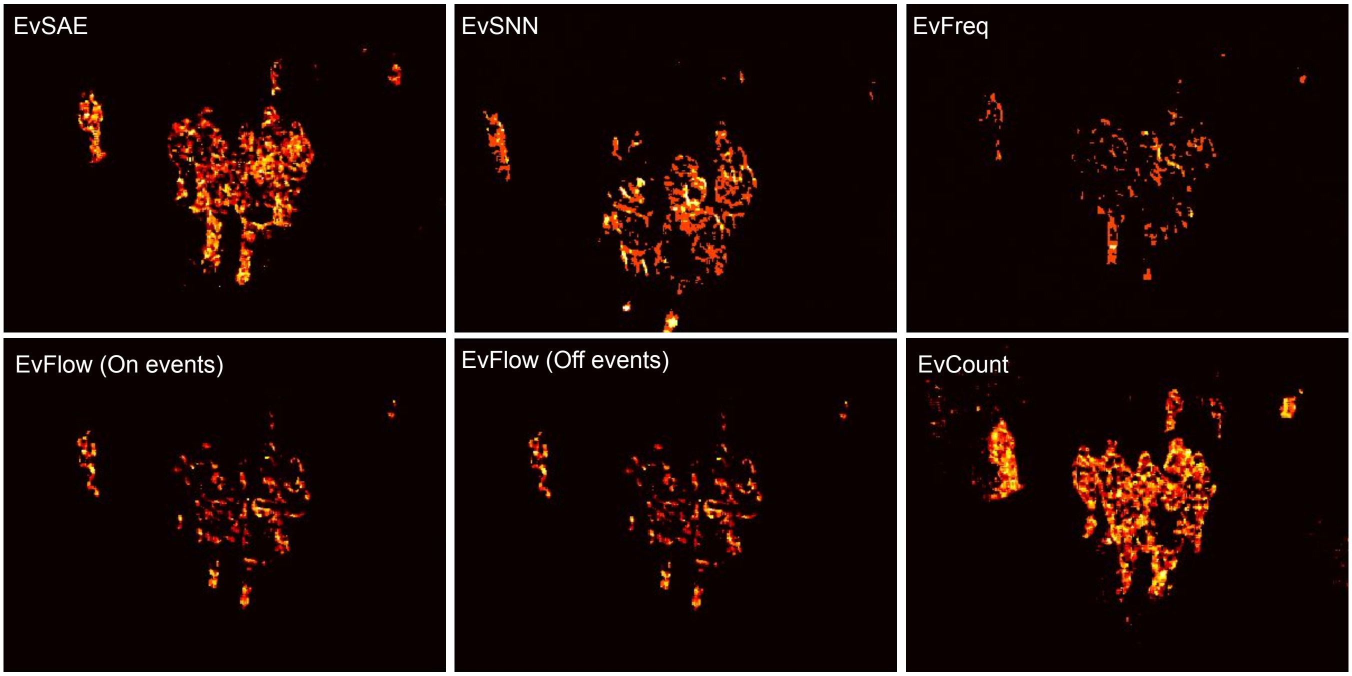

Fig. 4: Visualization of event data representations proposed in the literature.

MobileNet feature extraction has been applied to DL memory surfaces and other conventional memory surfaces. Support Vector Machine (SVM) has been trained for classifying activities (walking vs. other activities) on these MobileNet features. Our method was performing better than hand-crafted features across all the activities. However, to emphasize that the features learned by the proposed DL memory surface are superior to that of the hand-crafted memory surfaces, we have given the comparison (Fig. 5) in terms of different metrics such as recall, precision, and F1 measure on the activities which showed substantial improvement. This performance improvement is enabled because the proposed method has more parameters to learn during the training stage.

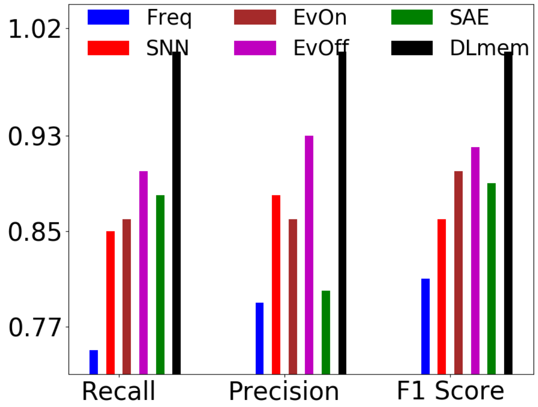

Fig. 5: Comparison of the proposed DL memory surface with hand-crafted memory surfaces in the context of activity recognition.

3 Computation Time Comparison with State-of-the-Art

An event camera's main advantage is low latency, which could be retained while processing if it involves asynchronous processing. However, it results in reduced accuracy. To trade latency for accuracy, researchers have proposed to accumulate events over a period of time. The proposed approach is one such approach where we have used a deep learning solution to learn representation from the data. As we may be sacrificing speed for improved accuracy, we also provide the computational complexity of the proposed approach with respect to non-deep learning approaches for a different number of input events. Table. 2 furnishes the computation time vs. the number of events, which is performed on a CPU (Intel i7 CPU, 64bits, 2.3GHz, and 64 GB of RAM) with GPU (GeForce RTX 1080 Ti). It can be seen from Table. 2 that the proposed approach is still a real-time solution because of the thin encoder network that we have proposed for DL memory surface generation.

Number of Events	SAE	SNN	Freq	EvOn	EvOff	DLmem
49806	0.008	22.49	0.011	0.025	0.020	0.055
54698	0.008	20.77	0.010	0.023	0.019	0.055
52387	0.008	18.71	0.013	0.030	0.023	0.055
50530	0.008	16.6	0.010	0.022	0.018	0.055
62310	0.011	14.9	0.013	0.028	0.024	0.055

TABLE 2: Comparison of run time (in ms) of the proposed DL memory surface network with other hand crafted methods whose details are provided in the paper.

REFERENCES

[1] Marco Ciccone, Andrea Romanoni, and Matteo Matteucci. A differentiable recurrent surface for asynchronous event-based data. *ECCV*, 2020.

[2] Calabrese E, Taverni G, and Awai Easthope C et. al. Dhp19: Dynamic vision sensor 3d human pose dataset. *CVPRW*, 2019.

[3] Gehrig and Daniel et al. End-to-end learning of representations for asynchronous event-based data. *Proceedings of the IEEE International Conference on Computer Vision*, 2019.

[4] Howard and Andrew G et al. Mobilenets: Efficient convolutional neural networks for mobile vision applications. *preprint arXiv:1704.04861*, 2017.

[5] A. Zhu, L. Yuan, K. Chaney, and K. Daniilidis. Ev-flownet: Self-supervised optical flow estimation for event-based cameras. *Proceedings of Robotics: Science and Systems*, 2018.

4 APPENDIX

This section provides explainable AI for DL memory surface, conditional GAN and sparse submanifold convolution.

4.1 Explainable AI for DL Memory Surface

This study performs an explainability-driven audit of our DL memory surface network via an analytical analysis. As Principal Component Analysis (PCA) has a strong theoretical background, we have attempted to demonstrate the relation of sparse activation regularization auto-encoder with PCA. For the purpose of this analytical proof, the notations used are as follows: $\mathbf{X} = [\mathbf{x}_1^T, \dots \mathbf{x}_N^T]^T, \widehat{\mathbf{X}} = [\widehat{\mathbf{x}}_1^T, \dots \widehat{\mathbf{x}}_N^T]^T$ are the input and output $N \times d$ matrices of the network, where $\mathbf{x}_i$ is the ith data point and N, d are the number of data points and dimension of the data point respectively and $\widehat{\mathbf{X}} = \mathbf{X}\mathbf{W}_1\mathbf{W}_2$. Let $\mathbf{W}_1$ and $\mathbf{W}_2$ be $d \times h$ and $h \times d$ weight matrices of encoder and decoder respectively. The loss term for a linear auto-encoder with a single hidden layer can be written as follows,

$$\min_{W_1} \|\mathbf{X} - \widehat{\mathbf{X}}\|_F^2 + \|\mathbf{X}\mathbf{W}_1 - \mathbf{S}\|_F^2 \tag{1}$$

The first term is the data term, and the second term is the sparsity term. Let $\mathbf{S}$ be $N \times h$ sparsity matrix, which defines a randomly generated sparsity matrix with a given sparsity factor. As analytical proof of the L_1 norm sparsity constraint is intractable, we have formulated it to minimize the L_2 norm between bottleneck layer activation and a generated sparsity matrix S.

Let us consider the first term. The first term is minimized when $\widehat{\mathbf{X}} = \mathbf{U}_r\Sigma_r\mathbf{V}_r^T$, where r is the rank of the matrix, $\mathbf{U}_r, \mathbf{V}_r$ are left singular vector and right singular vector matrices of $\mathbf{X}$ respectively with r non-zero columns corresponding to non-zero singular values. Σ_r is the singular value matrix of $\mathbf{X}$. This implies that $\widehat{\mathbf{X}} = \mathbf{X}\mathbf{W}_1\mathbf{W}_2 = \mathbf{U}_r\Sigma_r\mathbf{V}_r^T$, which could be split as $\mathbf{W}_2 = \mathbf{V}_r^T$ and $\mathbf{X}\mathbf{W}_1 = \mathbf{U}_r\Sigma_r$. Pre-multiplying by $\mathbf{X}^T$ and assuming the features are independent, which makes $(\mathbf{X}^T\mathbf{X})^{-1}$ invertible, $\mathbf{W}_1$ can be written as

$$\mathbf{W}_1 = (\mathbf{X}^T\mathbf{X})^{-1}\mathbf{X}^T\mathbf{U}_r\Sigma_r \tag{2}$$

Substituting SVD for $\mathbf{X}$ and using the fact that singular vector matrices are orthogonal, could be simplified to

$$\mathbf{W}_1 = \mathbf{V}(\mathbf{\Sigma}^T\mathbf{\Sigma})^{-1}\mathbf{\Sigma}^T\mathbf{U}^T\mathbf{U}_r\Sigma_r \tag{3}$$

Splitting $(\mathbf{\Sigma}^T\mathbf{\Sigma})^{-1}$ and using the fact that $\mathbf{U}^T\mathbf{U}_r = \mathbf{I}_r$, where $\mathbf{I}_r$ is $r \times r$ identity matrix, we get $\mathbf{W}_1 = V$. Now, considering the second term $\|\mathbf{X}\mathbf{W}_1 - \mathbf{S}\|_F^2$ which is equivalent of activation sparsity regularization term and pre and post multiplying with $\mathbf{U}$ (Frobenius norm does not change when mutiplied with orthogonal matrix), we get

$$\| \begin{bmatrix} \mathbf{U}_r^T \\ \mathbf{U}_{nr}^T \end{bmatrix} (\mathbf{X}\mathbf{W}_1 - \mathbf{S}) \begin{bmatrix} \mathbf{U}_r^T & \mathbf{U}_{nr}^T \end{bmatrix} \|_F^2 \tag{4}$$

Where $\mathbf{U}_{nr}$ is the part of $\mathbf{U}$ matrix with columns from r to N corresponding to zero singular values. Substituting the following SVD decomposition of $\mathbf{X}$ into the second term, we get the Eq. 6,

$$\begin{bmatrix} \mathbf{U}_r^T \\ \mathbf{U}_{nr}^T \end{bmatrix} \mathbf{X} = \begin{bmatrix} \mathbf{\Sigma}_r \\ 0 \end{bmatrix} \mathbf{V}^T \tag{5}$$

$$\| \left(\begin{bmatrix} \mathbf{\Sigma}_r \\ 0 \end{bmatrix} \mathbf{V}^T\mathbf{W}_1 - \begin{bmatrix} \mathbf{U}_r^T \\ \mathbf{U}_{nr}^T \end{bmatrix} \mathbf{S} \right) \begin{bmatrix} \mathbf{U}_r^T & \mathbf{U}_{nr}^T \end{bmatrix} \|_F^2 \tag{6}$$

This equation is minimized when the following equality is satisfied.

$$\begin{bmatrix} \mathbf{\Sigma}_r \\ 0 \end{bmatrix} \mathbf{V}^T\mathbf{W}_1 = \begin{bmatrix} \mathbf{U}_r^T \\ \mathbf{U}_{nr}^T \end{bmatrix} \mathbf{S} \tag{7}$$

This implies $\mathbf{\Sigma}_r\mathbf{V}^T\mathbf{W}_1 = \mathbf{U}_r^T\mathbf{S}$ and it leads to $\mathbf{W}_1 = \mathbf{V}\mathbf{\Sigma}_r^{-1}\mathbf{U}_r^T\mathbf{S}$. Now considering the data and sparsity term, we have seen that they are minimized when $\mathbf{W}_1$ is $\mathbf{V}$ and $\mathbf{V}\mathbf{\Sigma}_r^{-1}\mathbf{U}_r^T\mathbf{S}$ respectively. The bottleneck layer output $\mathbf{H} = \mathbf{X}\mathbf{W}_1$ is given by $\mathbf{X}\mathbf{V}$ and $\mathbf{U}_r\mathbf{U}_r^T\mathbf{S}$, which is obtained by substituting SVD decomposition of $\mathbf{X}$. It can be seen that the minimization of the data term leads to a solution similar to the PCA of the covariance matrix. The minimization of the sparsity term leads to a solution, which is the sparsity matrix's $(\mathbf{S})$ projection onto the data matrix's column space $(\mathbf{U}_r)$. The column space is constructed from left singular vectors that correspond to non-zero singular values.

4.2 Explainable AI for conditional GAN

The derivations for the working of GAN have been provided in [?]. Here, we are extending it to conditional GAN. Eq.**??** can be expanded as

$$\int_x \int_y \mathbb{P}_{dd}(x, y) \log[D(x, y)] + \int_x \int_y \mathbb{P}_{gd}(x, y) \log[1 - D(x, y)] \tag{8}$$

The optimal $D^*(x, y)$ is estimated by differentiating the above equation with respect to $D(x, y)$ and equating to zero,

$$D^*(x, y) = \frac{\mathbb{P}_{dd}(x, y)}{\mathbb{P}_{dd}(x, y) + \mathbb{P}_{gd}(x, y)} \tag{9}$$

Substituting the optimum value of D in the generator equation, and multiplying and dividing the terms inside $\log$ by 2 and using the fact $\log(AB) = \log(A) + \log(B)$, it yields

$$\min_{G} \quad \{-2\log(2) + KL\left[\mathbb{P}_{dd}(x,y)||\frac{\mathbb{P}_{dd}(x,y)+\mathbb{P}_{gd}(x,y)}{2}\right] \quad (10)$$
$$+KL\left[\mathbb{P}_{gd}(x,y)]\frac{\mathbb{P}_{dd}(x,y)+\mathbb{P}_{gd}(x,y)}{2}\right]\}$$

The second and third term together is nothing but $JSD(\mathbb{P}_{dd}(x,y)||\mathbb{P}_{gd}(x,y))$, where JSD is Jensen-Shannon Divergence. Thus, the objective function of G is minimized when $\mathbb{P}_{gd}(x,y) = \mathbb{P}_{dd}(x,y)$. This shows that cGAN learns the joint distribution of input and output to which it is exposed during training. Hence, it will not predict the output sequence of anomalies as the probability distribution of input-output pair of normal followed by anomaly or anomaly followed by anomaly have not been learned by cGAN.

4.3 Explainable AI for Submanifold Sparse Convolution

This section has tried to give an analytical analysis of the difference between general convolution and submanifold sparse convolution in $1d$ space.

Let $\mathbf{x}$ be a $1d$ signal, $\mathbf{k}$ be the $1d$ kernel and $\mathbf{C} = \mathbf{F}^h\mathbf{\Sigma F}$ (h denotes transpose of complex matrix) be the circulant convolution matrix formed from the kernel such that $\mathbf{y} = \mathbf{k}\circledast\mathbf{x} = \mathbf{Cx}$. $\mathbf{F} = [\mathbf{f_1}, \mathbf{f_2}, \ldots]$ and $\mathbf{\Sigma}$ are the Eigen vector and Eigen value matrices of $\mathbf{C}$ respectively. As $\mathbf{C}$ is circulant matrix, $\mathbf{F}$ is the Fourier matrix made up of Fourier bases $\mathbf{f_i}$ and $\mathbf{\Sigma}$ is the Fourier coefficients of $\mathbf{C}$. Submanifold sparse convolution can be written as $\mathbf{y_s} = \mathbf{MCx}$, where $\mathbf{M}$ is an Identity matrix with few of its rows (whose index correspond to the zero element of $\mathbf{y_s}$) made zero. Energy difference between the $\mathbf{y}$ and $\mathbf{y_s}$ is given as $||\mathbf{Cx} - \mathbf{MCx}||_F^2$. Substituting $\mathbf{C} = \mathbf{F^h\Sigma F}$ and pre-multiplying by $\mathbf{F}$ (multiplication by unitary matrix does not change the Frobenius norm), we obtain $||\mathbf{\Sigma Fx} - \mathbf{FMF^h\Sigma Fx}||_F^2$. Substituting $\mathbf{x_f} = \mathbf{Fx}$ (Fourier transform of $\mathbf{x}$) and $\mathbf{k_f} = \mathbf{\Sigma}$ (Fourier transform of $\mathbf{k}$), we obtain, $||\mathbf{k_f}\odot\mathbf{x_f} - \left(\mathbf{FMF^h}\right)\mathbf{k_f}\odot\mathbf{x_f}||_F^2$, where $\odot$ is element wise multiplication. When $\mathbf{FMF^h}$ is an identity matrix $\mathbf{I}$, general and submanifold sparse convolution are same. Hence, we need to analyse the term $\left(\mathbf{FMF^h}\right)\mathbf{k_f}\odot\mathbf{x_f}$. It can be written as follows,

$$\left(\mathbf{F}\begin{bmatrix}1 & 0 & 0 & \ldots\\ 0 & 1 & 0 & \ldots\\ 0 & 0 & 0 & \ldots\\ 0 & 0 & 0 & \ldots\\ \vdots & \vdots & \vdots & \vdots\end{bmatrix}\begin{bmatrix}\mathbf{f_1}^h\\ \mathbf{f_2}^h\\ \vdots\end{bmatrix}\mathbf{k_f}\right)\odot\mathbf{x_f} \quad (11)$$

Here it is assumed that the third and fourth elements of y_s are zero. The above equation can be written as,

$$\left(\begin{bmatrix}\mathbf{f_1} & \mathbf{f_2} & \mathbf{f_3} & \ldots\end{bmatrix}\begin{bmatrix}\mathbf{f_1}^h\mathbf{k_f}\\ \mathbf{f_2}^h\mathbf{k_f}\\ 0\\ \vdots\end{bmatrix}\right)\odot\mathbf{x_f} \quad (12)$$

The above equation can be simplified as $\left[\left(\mathbf{f_1}^h\mathbf{k_f}\right)\mathbf{f_1} + \left(\mathbf{f_1}^h\mathbf{k_f}\right)\mathbf{f_2} + (0)\,\mathbf{f_3} + \ldots\right]\odot\mathbf{x_f}$. $\left[\left(\mathbf{f_1}^h\mathbf{k_f}\right)\mathbf{f_1} + \left(\mathbf{f_1}^h\mathbf{k_f}\right)\mathbf{f_2} + (0)\,\mathbf{f_3} + \ldots\right]$ is $[k_1\mathbf{f_1} + k_2\mathbf{f_2} + 0\mathbf{f_3} + \ldots]$, where k_i is i^{th} element of the kernel $\mathbf{k}$. This can be expanded as follows

$$\begin{bmatrix}k_1 f_{11} + k_2 f_{21} + 0 f_{31} + \ldots\\ k_1 f_{12} + k_2 f_{22} + 0 f_{32} + \ldots\\ \vdots\end{bmatrix} \quad (13)$$

Where, f_{ik} is k^{th} element of ith bases. It could be seen that based on the sparsity of the data, certain elements of the Fourier bases are multiplied by 0. This will be the loss incurred by employing submanifold sparse convolution instead of general convolution.
